# Circular RNA circZFPM2 regulates cardiomyocyte hypertrophy and survival

**DOI:** 10.1007/s00395-024-01048-y

**Published:** 2024-04-19

**Authors:** Dimyana Neufeldt, Arne Schmidt, Elisa Mohr, Dongchao Lu, Shambhabi Chatterjee, Maximilian Fuchs, Ke Xiao, Wen Pan, Sarah Cushman, Christopher Jahn, Malte Juchem, Hannah Jill Hunkler, Giuseppe Cipriano, Bjarne Jürgens, Kevin Schmidt, Sonja Groß, Mira Jung, Jeannine Hoepfner, Natalie Weber, Roger Foo, Andreas Pich, Robert Zweigerdt, Theresia Kraft, Thomas Thum, Christian Bär

**Affiliations:** 1 Institute of Molecular and Translational Therapeutic Strategies, Hannover Medical School, Hannover, Germany; 2 Center for Translational Regenerative Medicine, Hannover Medical School, Hannover, Germany; 3 Fraunhofer Institute for Toxicology and Experimental Medicine (ITEM), Hannover, Germany; 4 Institute of Molecular and Cell Biology, A*Star, Singapore, Singapore; 5 Institute of Toxicology, Hannover Medical School, Hannover, Germany; 6 Core Facility Proteomics, Institute of Toxicology, Hannover, Germany; 7 Leibniz Research Laboratories for Biotechnology and Artificial Organs (LEBAO), Department of Cardiothoracic, Transplantation and Vascular Surgery, Hannover Medical School, Hannover, Germany; 8 Institute for Molecular and Cell Physiology, Hannover Medical School, Hannover, Germany

**Keywords:** Cardiac hypertrophy, Hypertrophic cardiomyopathy, Non-coding RNA, Circular RNA, Human induced pluripotent stem cell-derived cardiomyocytes, Heart organoids

## Abstract

Hypertrophic cardiomyopathy (HCM) constitutes the most common genetic cardiac disorder. However, current pharmacotherapeutics are mainly symptomatic and only partially address underlying molecular mechanisms. Circular RNAs (circRNAs) are a recently discovered class of non-coding RNAs and emerged as specific and powerful regulators of cellular functions. By performing global circRNA-specific next generation sequencing in cardiac tissue of patients with hypertrophic cardiomyopathy compared to healthy donors, we identified circZFPM2 (hsa_circ_0003380). CircZFPM2, which derives from the ZFPM2 gene locus, is a highly conserved regulatory circRNA that is strongly induced in HCM tissue. In vitro loss-of-function experiments were performed in neonatal rat cardiomyocytes, human induced pluripotent stem cell-derived cardiomyocytes (hiPSC-CMs), and HCM-patient-derived hiPSC-CMs. A knockdown of circZFPM2 was found to induce cardiomyocyte hypertrophy and compromise mitochondrial respiration, leading to an increased production of reactive oxygen species and apoptosis. In contrast, delivery of recombinant circZFPM2, packaged in lipid-nanoparticles or using AAV-based overexpression, rescued cardiomyocyte hypertrophic gene expression and promoted cell survival. Additionally, HCM-derived cardiac organoids exhibited improved contractility upon CM-specific overexpression of circZFPM2. Multi-Omics analysis further promoted our hypothesis, showing beneficial effects of circZFPM2 on cardiac contractility and mitochondrial function. Collectively, our data highlight that circZFPM2 serves as a promising target for the treatment of cardiac hypertrophy including HCM.

**Supplementary Information:**

The online version contains supplementary material available at 10.1007/s00395-024-01048-y.

## Introduction

Hypertrophic cardiomyopathy (HCM) is a complex genetic disorder of the heart that is characterized by commonly asymmetric cardiac hypertrophy, pronounced fibrosis and myocyte disarray [48]. In approximately 70% of the genotype-positive patients, a mutation in the *MYH7* or the *MYBPC3* gene, encoding the β-myosin heavy chain and myosin-binding protein C, respectively, causes the disease [2]. Despite similar genotypes, the phenotypes range from a relatively asymptomatic course, to the development of heart failure and even sudden cardiac death [3, 37]. With a prevalence of 1:200 to 1:500, HCM constitutes the most common primary genetic cardiovascular disorder [44]. Despite these high numbers, no specific genetic treatment options targeting the underlying molecular mechanisms exist as a standard of care, although there are currently few promising pre-clinical studies using gene therapy and gene editing approaches [7, 21, 52]. Current medications such as beta-blockers, calcium channel blockers or diuretics are inadequate and only achieve relief from symptoms [12]. Recently, the disease specific, first-in-class, cardiac myosin inhibitor mavacamten (MYK-461) successfully passed through a Phase 3 clinical trial and received market approval by the FDA and EMA for a specific subset of symptomatic HCM patients presenting a hypercontractility phenotype [51]. While, for this specific subset of patients, mavacamten might constitute a promising novel treatment option, for HCM patients not displaying hypercontractility, specific therapeutics are still scarce and of the utmost need.

In this regard, a growing number of studies in recent years have uncovered the essential regulatory functions of non-coding RNAs (ncRNAs), particularly microRNAs (miRNAs) or long ncRNAs (lncRNAs), in diverse biological processes and linked their derailed expression to various diseases [1, 9, 46, 61]. Due to their central role in pathogenesis, druggability, and cell type-specific expression, they are considered promising therapeutic targets in neurodegenerative diseases, cancer, and cardiovascular diseases [6, 22, 23, 56]. Circular RNAs (circRNAs) constitute the most recently discovered class of ncRNAs, which are formed through non-canonical splicing (also termed back-splicing) to form covalently closed RNA rings [11, 17]. They are abundant, highly conserved among species, and exert a variety of functions including sponging of miRNAs and RNA-binding proteins, serving as scaffolds and regulatory RNAs for protein complexes or as templates for the translation of micro-peptides [25, 45]. Due to the circular configuration, circRNAs exhibit an increased stability, which makes them suitable as biomarkers in addition to long lasting modulators of cellular functions compared to linear RNAs [24]. To date, several circRNAs were described with crucial regulatory functions in cardiovascular diseases, for instance: in myocardial infarction (CDR1-as), cardiac fibrosis (circHIPK3), atherosclerosis (cANRIL), cardiac senescence (circ-Foxo3), and doxorubicin-induced cardiomyopathy (circINSR) [5, 10, 15, 31, 59]. In the context of cardiac hypertrophy, the heart-related circRNA (HRCR) and the circRNA derived from the SLC8A1 gene (circSLC8A1) have been described [28, 57]. Mechanistically, they were suggested to exert their anti- and pro-hypertrophic traits through sponging miR-223 and miR-133a, respectively [28, 57]. Although, they represent the first circRNAs in cardiac hypertrophy, their impact specifically in HCM remains elusive.

Here, we employed global circRNA sequencing and specifically evaluated the role of circZFPM2 (has_circ_0003380, mmu_circ_0000597) as a protective circRNA in HCM. This circRNA derives from the ZFPM2/FOG2 locus and despite the well-known importance of its host gene in cardiogenesis, to our knowledge, the study at hand constitutes the first report about circZFPM2 in the cardiac field [53]. Our circRNA sequencing results revealed that circZFPM2 was upregulated in HCM patients and by performing loss- and gain-of-function experiments, we elucidated the potential of this circRNA as a novel therapeutic approach in HCM.

## Materials and methods

### Heart tissue sampling

Heart tissue samples for RNA sequencing were obtained from 5 HCM patients after myectomy and 5 healthy donors (Supplemental Table 1). For qPCR validation, heart tissue samples from 18 HCM patients and 18 healthy donors were utilized (Supplemental Table 2). This study was conducted with the approval of the institutional ethics committee of the Hannover Medical School, Germany, and in accordance with the guidelines from the declaration of Helsinki and its amendments or comparable ethical standards. For details, see Supplementary material online.

### Cell culture, treatments and cellular assays

Neonatal rat cardiomyocytes (NRCMs), mouse atrial-like cardiomyocytes (HL-1 cells), human induced pluripotent stem cells (hiPSCs) (hHSC_Iso4_ADCF_SeV-iPS2, alternative name: MHHi001-A) [18], HCM patient-derived iPSCs [32] and human embryonic kidney cells (HEK293) were cultured according to standard protocols. HiPSCs were differentiated into cardiomyocytes by modulating the Wnt pathway as previously described [27]. Cell treatments, transfections, and cellular assays were conducted as indicated in the Supplementary material online.

### Modulation of circRNA expression

Mouse, rat and human circZFPM2 were knocked down using a siRNA-mediated strategy. For overexpression experiments in NRCMs, the rodent circZFPM2 sequence with flanking circularization elements was cloned into the AAV MCS 1.3 plasmid using the restriction enzymes EcoRI and XbaI. For human overexpression studies, circZFPM2 was in vitro transcribed using the RiboMax large RNA production system (Promega) with a modified protocol. For details, see Supplementary material online.

### Gene expression analysis

Total RNA was isolated with QIAzol (Qiagen) according to the manufacturer’s protocol, of which 500–1000 ng were reverse transcribed using the Biozym cDNA Synthesis Kit with random hexamer primers according to the manufacturer’s protocol and assessed by quantitative real-time PCR (qRT-PCR) (Absolute Blue qPCR SYBR Green Mix, Thermo Scientific). For details, see Supplementary material online.

### Statistics

Data is presented as mean ± SD. For all results, statistics were calculated using GraphPad Prism software. Significant differences between 2 groups with < 6 data points per group were calculated using the non-parametric unpaired 2-tailed t test, while the parametric test was used for groups with > 6 data points. The non-parametric 1-way ANOVA with post hoc test was used for ≥ 3 groups with < 6 data points and parametric test with > 6 data points per group. *p < 0.05* was considered as statistically significant.

## Results

### Identification and validation of circZFPM2 dysregulation in HCM

**Fig. 1:**
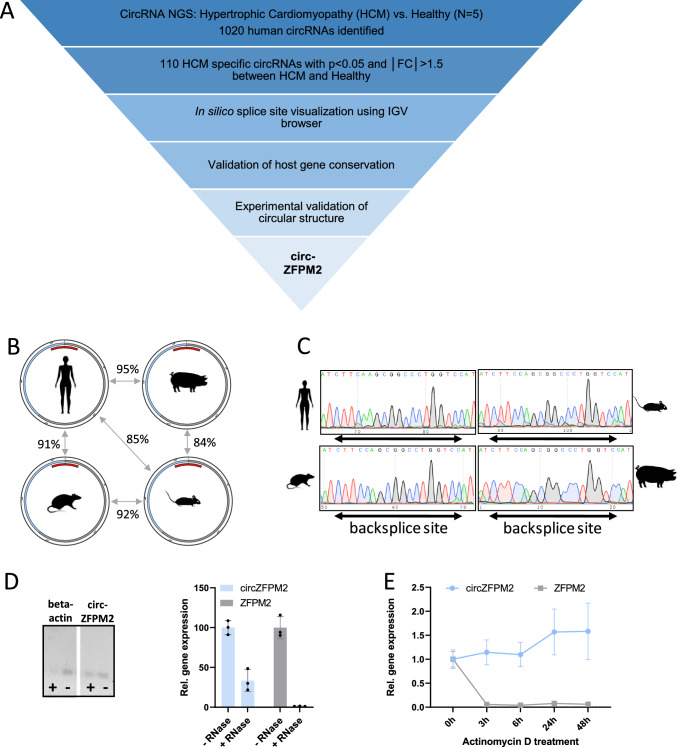
Identification and validation of conserved circRNAs in HCM identifies circZFPM2. **A** Schematic representation of the circRNA candidate selection pipeline. IGV, Integrative Genomics Viewer; NGS, Next Generation Sequencing. **B** Degree of sequence conservation (% nucleotide identity) of circZFPM2 between human, pig, rat and mouse. Blue and grey depict ZFPM2 Exons 2 and 3 forming the circle. Red denotes the backsplicing region. **C** Sanger sequencing results of conserved backsplice regions of circZFPM2 in human, pig, mouse and rat amplified with divergent primers. **D** Representative gel images of RT-PCR products and RT-qPCR results of RNase R treated murine cardiac RNA (n = 3 mice). **E** Relative expression of circZFPM2 and ZFPM2 in NRCMs after actinomycin D treatment (n = 3 wells/group). Data are means ± SD

To identify circRNAs that participate in the pathogenesis of HCM, we performed global circRNA profiling in cardiac tissue from healthy donors and HCM patients (Supplemental Table 1). 1020 circRNAs were detected, of which 110 (Supplemental Table 3) were significantly differentially expressed (p < 0.05 and fold change > 1.5) between the two groups (Fig. 1A). By conducting in silico and experimental validation of the 30 most differentially expressed and most abundant circRNAs, we identified circZFPM2 as the most promising candidate for further validation. In detail, in silico validation was performed using the integrative genomics viewer (Supplementary Figure S1A). CircRNAs for which the sequencing reads did not cover the backsplice site, were excluded. Furthermore, circZFPM2 was the only candidate, which was not detectable in hiPSCs, but which became abundantly expressed during the differentiation into hiPSC-CMs, suggesting CM relevant functions (Supplementary Figure S1B). CircZFPM2 derives from exon 2 and 3 of the ZFPM2 locus, which codes for a zinc finger protein that is essential for cardiogenesis and hematopoiesis in mammals [8, 53]. CircZFPM2 is annotated in the human and mouse genome (has_circ_0003380 and mmu_circ_0000597; circBase.org). By performing sequence alignment using NCBI standard nucleotide BLAST, we further detected a high degree of sequence homology in the rat and pig genome (Fig. 1B). We experimentally validated the correct backsplice regions in these four species by RT-qPCR using specific divergent primers and Sanger sequencing (Fig. 1C). Increased resistance towards RNase R treatment of RNA isolated from murine left ventricular tissue revealed circular RNA configuration (Fig. 1D). This was corroborated by prolonged stability of circZFPM2 compared to linear ZFPM2 after treatment of NRCMs with the transcription inhibitor actinomycin D (Fig. 1E).

In summary, we identified circZFPM2 as a *bona fide* circRNA regulated in HCM patients. Its high conservation suggests an important regulatory role and enables translational studies in various species.

### CircZFPM2 is regulated in HCM

**Fig. 2:**
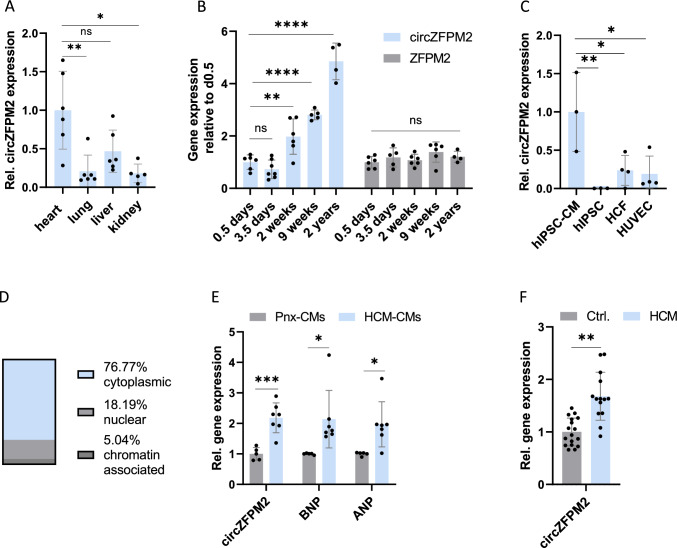
CircZFPM2 is a cardiomyocyte-enriched, cytoplasmic circRNA that is regulated in HCM.** A** Relative circZFPM2 expression in a murine organ panel. Non-parametric one-way ANOVA was performed to calculate significances (n = 5–6 mice). **B** Relative expression of circZFPM2 and ZFPM2 in a timeline of murine cardiac development. Parametric one-way ANOVA was performed to calculate significances to 0.5 days of each group (n = 4–6 mice/group). **C** Relative circZFPM2 expression in different human cell lines. Non-parametric one-way ANOVA was performed to calculate significances (n = 3–4 wells/group). hiPSC, human induced pluripotent stem cell; hiPSC-CM, hiPSC-derived cardiomyocyte; HCF, human cardiac fibroblast; HUVEC, human umbilical vein endothelial cell. **D** CircZFPM2 expression in subcellular fractions of hiPSC-CMs (n = 9 fractionations). **E** Relative expression of circZFPM2 and hypertrophy marker genes in CMs derived from healthy iPSCs (Pnx-CMs) and from HCM patient-derived hiPSCs. Parametric t-test was performed to calculate significances (n = 5–7 wells/group). HCM, hypertrophic cardiomyopathy. **F** Relative expression of circZFPM2 and ZFPM2 in HCM patient-derived cardiac tissue compared to healthy controls. Parametric t-test was performed to calculate significances (n = 18 subjects/group). Data are means ± SD. *p < 0.05; **p < 0.01; ***p < 0.001; ****p < 0.0001

For further characterization of circZFPM2, we evaluated its expression profile in a mouse organ panel revealing highest expression in the heart (Fig. 2A), which gradually increases with age, independent of the linear ZFPM2 (Fig. 2B). In a collection of different human cell types, circZFPM2 is most abundant in hiPSC-CMs (Fig. 2C), while on the subcellular level, it is primarily localized in the cytoplasm (Fig. 2D).

Next, we aimed to validate the initial RNA sequencing data revealing a 2.5-fold upregulation of circZFPM2 in human HCM heart tissue, in further HCM models. First, we utilized HCM patient-derived iPSC-CMs carrying the disease causing point mutation R723G in the *MYH7* gene [47]. Consistently, circZFPM2 was significantly upregulated in the diseased cells compared to the wild type control hiPSC-CMs, which is in line with increased expression of the hypertrophy marker genes *BNP* and *ANP* (Fig. 2E). Importantly, we confirmed the significantly increased expression of circZFPM2 in independent biopsy material from HCM patients compared to healthy donor heart tissue (Fig. 2F). Interestingly, the expression of circZFPM2 in other in vivo and in vitro cardiac disease models such as myocardial infarction (MI), hypoxia exposed NRCMs or TGFβ treated human cardiac fibroblasts, remained unchanged, suggesting HCM-specific regulation of this circRNA candidate (Supplementary Figure S2 A–C).

In conclusion, circZFPM2 is a cardiomyocyte-enriched, cytoplasmic circRNA that is upregulated in HCM.

### CircZFPM2 silencing promotes hypertrophy, metabolic, and cytotoxic effects in neonatal primary rat cardiomyocytes

**Fig. 3:**
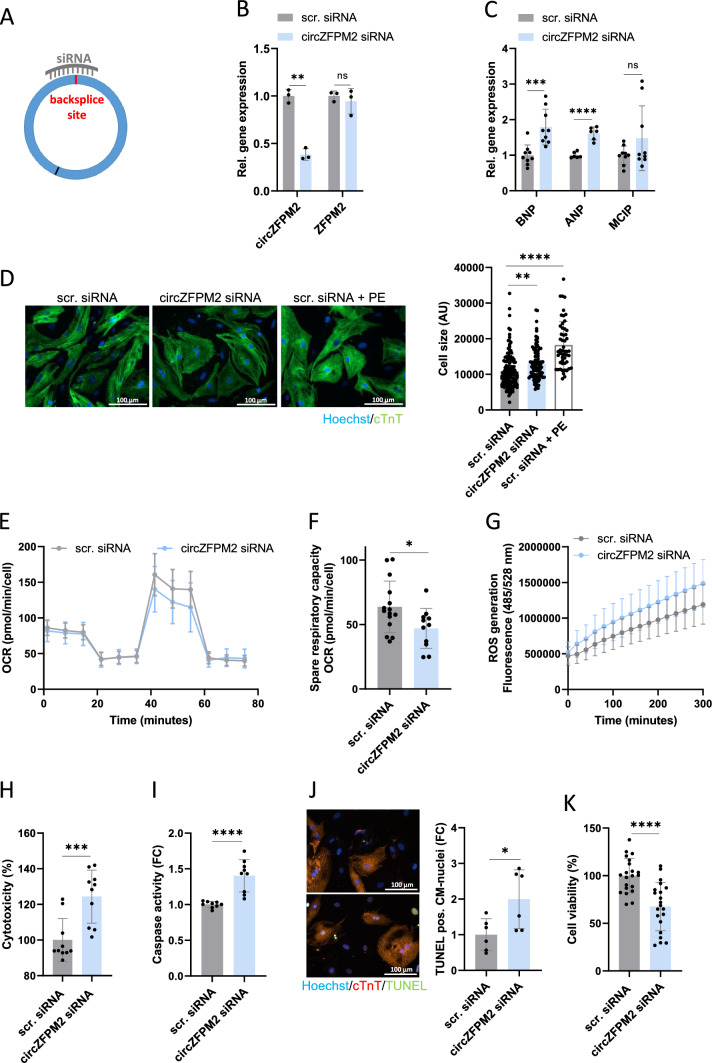
CircZFPM2 silencing impairs NRCM function. **A** Schematic representation of a backsplice site specific siRNA. **B** Relative expression of circZFPM2 and ZFPM2 in circZFPM2 siRNA and scrambled siRNA (scr. siRNA) treated NRCMs. Non-parametric t-test was performed to calculate significances (n = 3 wells/group) **C** Relative expression of hypertrophy marker genes in circZFPM2 siRNA and scrambled siRNA (scr. siRNA) treated NRCMs. Parametric t-test was performed to calculate significances (n = 6–9 wells/group). **D** Representative images and cell size measurement of scrambled siRNA (scr. siRNA), circZFPM2 siRNA and scrambled siRNA plus 100 µM phenylephrine (scr. siRNA + PE) treated NRCMs after Hoechst and cTnT (cardiac troponin T) staining in arbitrary units (AU). Parametric one-way ANOVA was performed to calculate significances to scrambled siRNA control (n ≥ 65 cells/group). **E** Oxygen consumption rate (OCR) of scrambled siRNA (scr. siRNA) and circZFPM2 siRNA treated NRCMs, measured by Seahorse XF Mito Stress Test (n = 11–16 wells/group). **F** Spare respiratory capacity of scrambled siRNA (scr. siRNA) and circZFPM2 siRNA treated NRCMs, measured by Seahorse XF Mito Stress Test. Parametric t-test was performed to calculate significances (n = 11–15 wells/group). **G** Production of reactive oxygen species (ROS) of scrambled siRNA (scr. siRNA) and circZFPM2 siRNA treated NRCMs, measured by the DCFDA/H2DCFDA—Cellular ROS Assay Kit. Data are means ± SEM (n = 6 wells/group). **H** Percentage of cytotoxicity of scrambled siRNA (scr. siRNA) and circZFPM2 siRNA treated NRCMs, measured with the CytoTox 96 Non-Radioactive Cytotoxicity Assay kit. Parametric t-test was performed to calculate significances (n = 10 wells/group). **I** Fold change (FC) of caspase activity of scrambled siRNA (scr. siRNA) and circZFPM2 siRNA treated NRCMs, measured with the Caspase-Glo 3/7 kit. Parametric t-test was performed to calculate significances (n = 9 wells/group). **J** Representative images and fold change (FC) of TUNEL positive nuclei of cTnT positive NRCMs after scrambled siRNA (scr. siRNA) and circZFPM2 siRNA treatment. Parametric t-test was performed to calculate significances (n = 6 wells/group). **K** Percentage of cell viability of scrambled siRNA (scr. siRNA) and circZFPM2 siRNA treated NRCMs, measured with the Cell Proliferation Reagent WST-1 kit. Parametric t-test was performed to calculate significances (n = 21 wells/group). Data are means ± SD. *p < 0.05; **p < 0.01; ***p < 0.001; ****p < 0.0001; ns = not significant. FC, fold change

To elucidate whether circZFPM2 (dys)regulation has functional consequences, we first performed loss-of-function experiments in NRCMs. We designed a specific siRNA targeting the backsplice region of circZFPM2 resulting in efficient knockdown without affecting the expression of the linear host gene (Fig. 3A, B).

Knockdown of circZFPM2 led to significantly increased expression of the hypertrophy marker genes *BNP* and *ANP* (not significant for *MCIP*) (Fig. 3C). In line with these findings, lack of circZFPM2 induced cellular hypertrophy in NRCMs compared to the scrambled siRNA treated control cells (Fig. 3D).

Since HCM is associated with altered cell metabolism, we conducted a Seahorse XF Cell Mito Stress assay after circZFPM2 knockdown to test for a potential mitochondrial functional impairment [42]. Indeed, the oxygen consumption rate (OCR) of NRCMs was reduced after circZFPM2 silencing, most pronounced was the spare respiratory capacity (Fig. 3E, F). Interestingly, either as a cause of or as a consequence for mitochondrial impairment, we detected an elevated production of reactive oxygen species (ROS) after circZFPM2 knockdown (Fig. 3G). Consequently, circZFPM2 knockdown increased cytotoxicity (Fig. 3H), caspase activity (Fig. 3I) and the number of apoptotic cells (TUNEL^+^; Fig. 3J), collectively leading to decreased cell viability (Fig. 3K) of NRCMs.

### CircZFPM2 overexpression rescues cardiomyocyte function in NRCMs

**Fig. 4:**
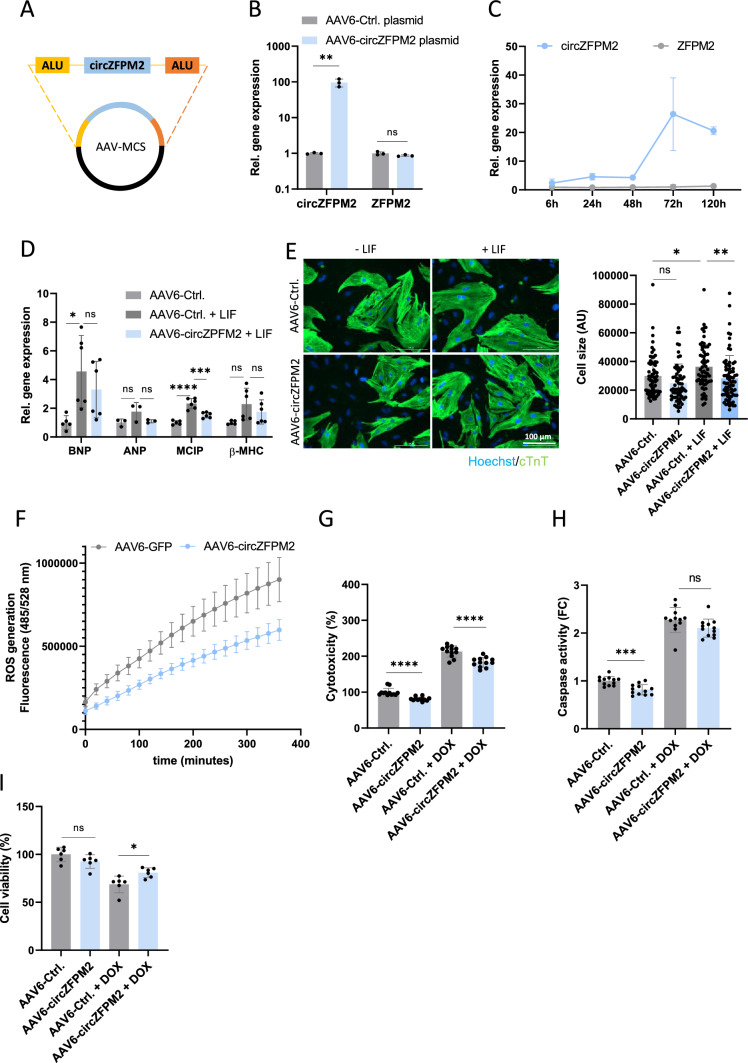
CircZFPM2 overexpression in NRCMs rescues cardiomyocyte function. **A** Schematic representation of the circZFPM2 overexpression cassette in AAV6 expression plasmid. **B** Relative expression of circZFPM2 and ZFPM2 in HL-1 cells transfected with the AAV6-circZFPM2 overexpression plasmid compared to the control (AAV6-Ctrl) plasmid. Non-parametric t-test was performed to calculate significances (n = 3 wells/group). **C** Timeline of relative circZFPM2 and ZFPM2 expression in NRCMs transduced with AAV6-circZFPM2 compared to AAV6-Ctrl. (n = 3–4 wells/group). **D** Relative expression of hypertrophy marker genes in NRCMs transduced with AAV6-circZFPM2 compared to AAV6-Ctrl., with and without hypertrophic stimulus (48 h LIF (leukemia inhibitor factor)). Parametric one-way ANOVA was performed to calculate significances to the respective AAV6-Ctrl. (n = 3–6 wells/group). **E** Representative images and cell size measurement of NRCMs transduced with AAV6-circZFPM2 compared to AAV6-Ctrl., with and without hypertrophic stimulus (48 h LIF treatment) in arbitrary units (AU). One-way ANOVA was performed to calculate significances (n > 200 CMs from 4 wells/group). **F** Production of reactive oxygen species (ROS) of NRCMs transduced with AAV6-circZFPM2 compared to AAV6-Ctrl., measured by the DCFDA/H2DCFDA–Cellular ROS Assay Kit. Data are means ± SEM (n = 6 wells/group). **G** Percentage of cytotoxicity of NRCMs transduced with AAV6-circZFPM2 compared to AAV6-Ctrl., with and without doxorubicin (DOX, 1 µM) treatment, measured with the CytoTox 96 Non-Radioactive Cytotoxicity Assay kit. Parametric t-test was performed to calculate significances (n = 12 wells/group). **H** Fold change (FC) of caspase activity of NRCMs transduced with AAV6-circZFPM2 compared to AAV6-Ctrl., with and without doxorubicin (DOX, 1 µM) treatment, measured with the Caspase-Glo 3/7 kit. Parametric t-test was performed to calculate significances (n = 12 wells/group). **I** Percentage of cell viability of NRCMs transduced with AAV6-circZFPM2 compared to AAV6-Ctrl., with and without doxorubicin (DOX, 1 µM) treatment, measured with the Cell Proliferation Reagent WST-1 kit. Parametric t-test was performed to calculate significances (n = 6 wells/group). Data are means ± SD. *p < 0.05; **p < 0.01; ***p < 0.001; ****p < 0.0001; ns = not significant

To test whether circZFPM2 overexpression might exert opposite, i.e. beneficial effects compared to the knockdown, we designed a circZFPM2 overexpression cassette in an AAV backbone, harboring the circRNA sequence flanked by intronic sequences containing inverted repeats (ALU elements) to facilitate the circularization (Fig. 4A) [31]. The transfection of this plasmid into the cardiomyocyte cell line HL-1 was already sufficient to induce a significant upregulation of circZFPM2, without influencing the linear counterpart (Fig. 4B). We next produced and transduced AAV6-circZFPM2 viral particles which led to a robust and stable overexpression of circZFPM2 in NRCMs, while the host gene expression remained unchanged (Fig. 4C).

In order to test circZFPM2 effects in combination with a hypertrophic stimulus, we treated NRCMs with leukemia inhibitory factor (LIF). Simultaneous overexpression of circZFPM2 blunted the expression of hypertrophy marker genes (significant only for *MCIP*) (Fig. 4D). Nevertheless, circZFPM2 overexpression fully rescued the LIF induced cellular hypertrophy to control levels (Fig. 4E).

In contrast to the previous knockdown experiments, circZFPM2 overexpression reduced cellular ROS production (Fig. 4F). Already at baseline, AAV6-circZFPM2 treatment reduced cytotoxicity and caspase activity. When NRCMs were additionally exposed to the cardiotoxic anti-cancer agent doxorubicin (0.25 µM), circZFPM2 partially rescued the cytotoxic effects and cell viability (Fig. 4G–I) [35].

In conclusion, overexpression of circZFPM2 rescues NRCM function including reduced hypertrophy, improved mitochondrial performance, and cell viability.

### CircZFPM2 is functionally conserved in human cardiomyocytes

**Fig. 5:**
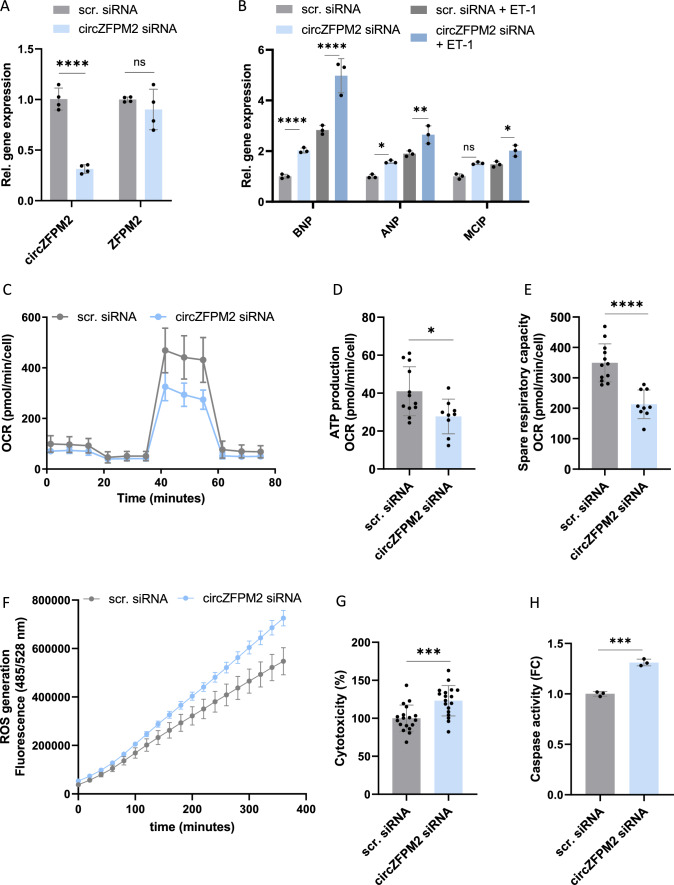
CircZFPM2 silencing impairs hiPSC-CMs function. **A** Relative expression of circZFPM2 and ZFPM2 in circZFPM2 siRNA and scrambled siRNA (scr. siRNA) treated hiPSC-CMs. Non-parametric t-test was performed to calculate significances (n = 4 wells/group). **B** Relative expression of hypertrophy marker genes in circZFPM2 siRNA and scrambled siRNA (scr. siRNA) treated hiPSC-CMs. Non-parametric one-way ANOVA was performed to calculate significances to the respective AAV6-Ctrl. (n = 3 wells/group). **C** Oxygen consumption rate (OCR) of scrambled siRNA (scr. siRNA) and circZFPM2 siRNA treated hiPSC-CMs, measured by Seahorse XF Mito Stress Test (n = 9–12 wells/group). **D** ATP production of scrambled siRNA (scr. siRNA) and circZFPM2 siRNA treated hiPSC-CMs, measured by Seahorse XF Mito Stress Test. Parametric t-test was performed to calculate significances (n = 9–12 wells/group). **E** Spare respiratory capacity of scrambled siRNA (scr. siRNA) and circZFPM2 siRNA treated hiPSC-CMs, measured by Seahorse XF Mito Stress Test. Parametric t-test was performed to calculate significances (n = 9–12 wells/group). **F** Production of reactive oxygen species (ROS) of scrambled siRNA (scr. siRNA) and circZFPM2 siRNA treated hiPSC-CMs, measured by the DCFDA/H2DCFDA – Cellular ROS Assay Kit. Data are means ± SEM. (n = 6 wells/group). **G** Percentage of cytotoxicity of scrambled siRNA (scr. siRNA) and circZFPM2 siRNA treated hiPSC-CMs, measured with the CytoTox 96 Non-Radioactive Cytotoxicity Assay kit. Parametric t-test was performed to calculate significances (n = 18 wells/group). **H** Fold change (FC) of caspase activity of scrambled siRNA (scr. siRNA) and circZFPM2 siRNA treated hiPSC-CMs, measured with the Caspase-Glo 3/7 kit. Non-parametric t-test was performed to calculate significances (n = 3 wells/group). Data are means ± SD. *p < 0.05; **p < 0.01; ***p < 0.001; ****p < 0.0001; ns = not significant

To extend the clinical value of our studies, we conducted loss- and gain-of-function experiments in human iPSC-CMs [34]. Similar to the experiments performed in NRCMs, we designed species-specific siRNAs targeting the backsplice site to knock down the human circZFPM2, without affecting host gene expression (Fig. 5A). To induce hypertrophy in hiPSC-CMs, cells were treated with ET-1 instead of PE or LIF, as performed for NRCMs. The pro-hypertrophic stimulation with ET-1 caused the most consistent hypertrophic response in hiPSC-CMs compared to PE or LIF, as evident by increased expression levels of several hypertrophic marker genes. CircZFPM2 silencing increased hypertrophic marker gene expression at baseline and showed additive effects when cells were treated with pro-hypertrophic stimuli (Fig. 5B). Metabolic impairment after circZFPM2 knockdown, as determined by Seahorse XF Cell Mito Stress assay, was even more pronounced in the human cardiomyocytes compared to rodents (Fig. 5C–E). Congruent with the effects seen in NRCMs, ROS production was enhanced after siRNA transfection (Fig. 5F) and, in turn, cytotoxicity and caspase activity were both significantly increased in hiPSC-CMs (Fig. 5G, H).

In summary, these experiments suggest that circZFPM2 is not only sequence, but also functionally conserved across species including in humans.

### In vitro transcribed circZFPM2 exerts beneficial effects in hiPSC-CMs

**Fig. 6:**
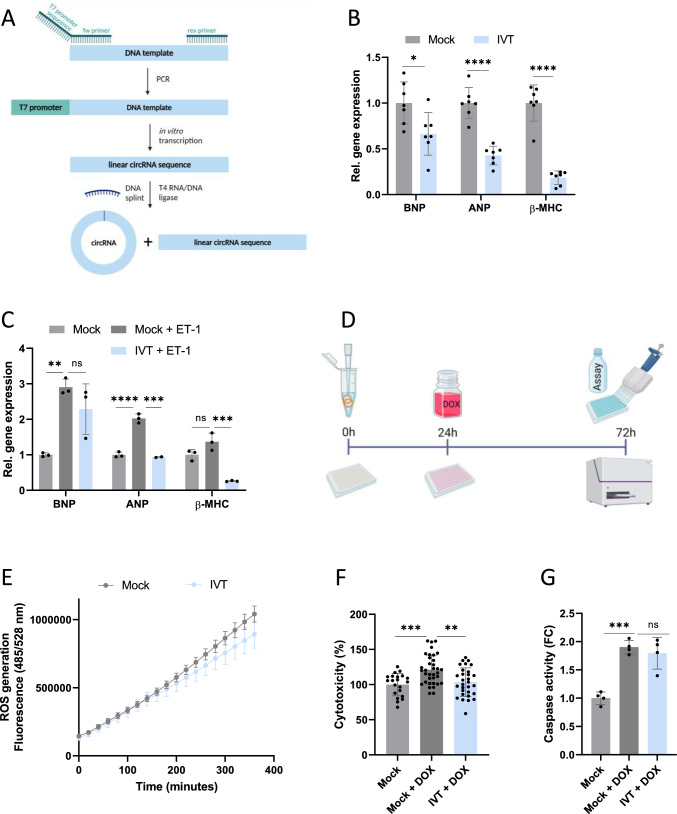
In vitro transcribed circZFPM2 rescues function of hiPSC-CMs. **A** Strategy of in vitro transcription of circRNAs. **B** Relative expression of hypertrophy marker genes in hiPSC-CMs transfected with the in vitro transcribed (IVT) circZFPM2 compared to the Mock-Ctrl. (only transfection reagent). Parametric t-test was performed to calculate significances to the respective Mock-control (n = 6 wells/group). **C** Relative expression of hypertrophy marker genes in hiPSC-CMs transfected with the IVT circZFPM2 compared to the Mock-Ctrl. after induction of hypertrophy with endothelin-1 (ET-1, 50 nM). Non-parametric one-way ANOVA was performed to calculate significances to the respective “Mock + ET-1”-control (n = 2–3 wells/group). **D** Schematic representation of the preventive IVT circZFPM2 treatment; created with BioRender.com. **E** Production of reactive oxygen species (ROS) of hiPSC-CMs transfected with the IVT circZFPM2 compared to Mock-Ctrl., measured by the DCFDA/H2DCFDA – Cellular ROS Assay Kit. Data are means ± SEM. (n = 6 wells/group). **F** Percentage of cytotoxicity of doxorubicin (DOX, 1 µM) treated hiPSC-CMs transfected with the IVT circZFPM2 compared to Mock-Ctrl., measured with the CytoTox 96 Non-Radioactive Cytotoxicity Assay kit. Parametric one-way ANOVA was performed to calculate significances to the “Mock + DOX”-group (n = 21–24 wells/group). **G** Fold change (FC) of caspase activity of doxorubicin (DOX, 1 µM) treated hiPSC-CMs transfected with the IVT circZFPM2 compared to Mock-Ctrl., measured with the Caspase-Glo 3/7 kit. Non-parametric one-way ANOVA was performed to calculate significances to the “Mock + DOX”-group (n = 4 wells/group). Data are means ± SD. *p < 0.05; **p < 0.01; ***p < 0.001; ****p < 0.0001; ns = not significant

To provide further translational evidence, we next aimed to investigate therapeutic effects of circZFPM2 by means of in vitro transcribed (IVT) circRNA treatment. We transcribed the linear human circZFPM2 sequence in vitro and circularized the RNA with the help of a backsplice site-complementary DNA splint and the T4 DNA ligase (Fig. 6A). This yielded IVT circZFPM2 of the correct size and with the correct backsplice sequence, which consequently showed robust, stable, and dose-dependent expression levels after transfection into hiPSC-CMs (Supplementary Figure S3A–D).

Functionally, IVT circZFPM2 significantly reduced hypertrophy marker gene expression at baseline and after hypertrophy induction (Fig. 6B, C). For the assessment of metabolic alterations and cell fitness, we stressed hiPSC-CMs with doxorubicin (1 µM) 24 h after IVT circZFPM2 transfection and incubated for additional 48 h before cell assays were performed (Fig. 6D). Similar to viral overexpression in rodents, ROS production was decreased through IVT circZFPM2 treatment (Fig. 6E). While cytotoxicity was significantly rescued, caspase activity remained unchanged (Fig. 6F, G).

These results suggest that treatment with IVT circZFPM2 confers comparable cardioprotective effects in hiPSC-CMs compared with AAV6-mediated overexpression in NRCMs.

### CircZFPM2 overexpression partially reverses the hypertrophic phenotype of HCM-CMs

To investigate whether the encouraging results gained from NRCMs and wild type hiPSC-CMs are translatable to the context of HCM, we performed gain- and loss-of-function experiments in HCM patient-derived iPSC-CMs with the R723G mutation in *MYH7*.

**Fig. 7:**
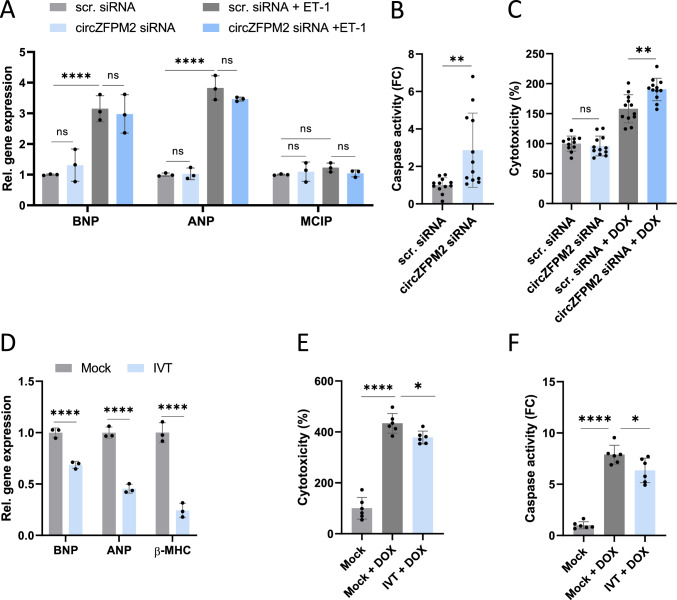
CircZFPM2 silencing impairs cell health of HCM-CMs, while IVT circZFPM2 rescues cardiomyocyte function. **A** Relative expression of hypertrophy marker genes in circZFPM2 siRNA and scrambled siRNA (scr. siRNA) treated HCM-CMs with and without hypertrophic stimulus (endothelin-1, ET-1). Non-parametric one-way ANOVA was performed to calculate significances to the respective control (n = 3 wells/group). **B** Fold change (FC) of caspase activity of scrambled siRNA (scr. siRNA) and circZFPM2 siRNA treated HCM-CMs, measured with the Caspase-Glo 3/7 kit. Parametric t-test was performed to calculate significances (n = 12 wells/group). **C** Percentage of cytotoxicity of scrambled siRNA (scr. siRNA) and circZFPM2 siRNA treated hiPSC-CMs, with and without doxorubicin (DOX, 1 µM) stress, measured with the CytoTox 96 Non-Radioactive Cytotoxicity Assay kit. Parametric t-test was performed to calculate significances (n = 12 wells/group). **D** Relative expression of hypertrophy marker genes in HCM-CMs transfected with the in vitro transcribed (IVT) circZFPM2 compared to the Mock-Ctrl. Non-parametric t-test was performed to calculate significances to the respective Mock-control (n = 3 wells/group). **E** Percentage of cytotoxicity of doxorubicin (DOX, 1 µM) treated HCM-CMs transfected with the IVT circZFPM2 compared to Mock-Ctrl., measured with the CytoTox 96 Non-Radioactive Cytotoxicity Assay kit. Parametric one-way ANOVA was performed to calculate significances to the “Mock + DOX”-group (n = 6 wells/group). **F** Fold change of caspase activity of doxorubicin (DOX, 1 µM) HCM-CMs transfected with the IVT circZFPM2 compared to Mock-Ctrl., measured with the Caspase-Glo 3/7 kit. Parametric one-way ANOVA was performed to calculate significances to the “Mock + DOX”-group (n = 6 wells/group). Data are means ± SD. *p < 0.05; ***p < 0.001; ****p < 0.0001; ns = not significant

First, we performed an siRNA-mediated knockdown of circZFPM2, which did not lead to increased hypertrophic marker gene expression, neither at baseline, nor in combination with pro-hypertrophic stimuli (Fig. 7A). Nevertheless, circZFPM2 silencing elevated caspase activity under basal condition (Fig. 7B) and further increased the cytotoxic effects of doxorubicin (Fig. 7C).

Strikingly, treatment of HCM-CMs with IVT circZFPM2 led to a drastic reduction of hypertrophic marker genes (Fig. 7D) concomitant with partially rescued cytotoxicity and caspase activity (Fig. 7E, F).

In line with our AAV6 in vitro overexpression model in non-diseased CMs, transfection of IVT circZFPM2 additionally reduced both apoptosis and cellular hypertrophy, while increasing cell survival in HCM-derived hiPSC-CMs.

### Human cardiac organoids exhibit improved contractility upon circZFPM2 overexpression

Furthermore, in order to demonstrate a more translational therapeutic approach by overexpressing circZFPM2 in a multi cellular 3D cardiac model, HCM-CMs were transduced with AAV6-circZFPM2 or a AAV6-GFP control. Together with ADSCs, HCFs, and HUVECs, the transduced HCM-CMs were assembled into HCM human cardiac organoids (HCM-hCOs) to investigate whether overexpression could protect against cardiac dysfunction using both a structural and functional miniature 3D cardiac replication of HCM.

**Fig. 8:**
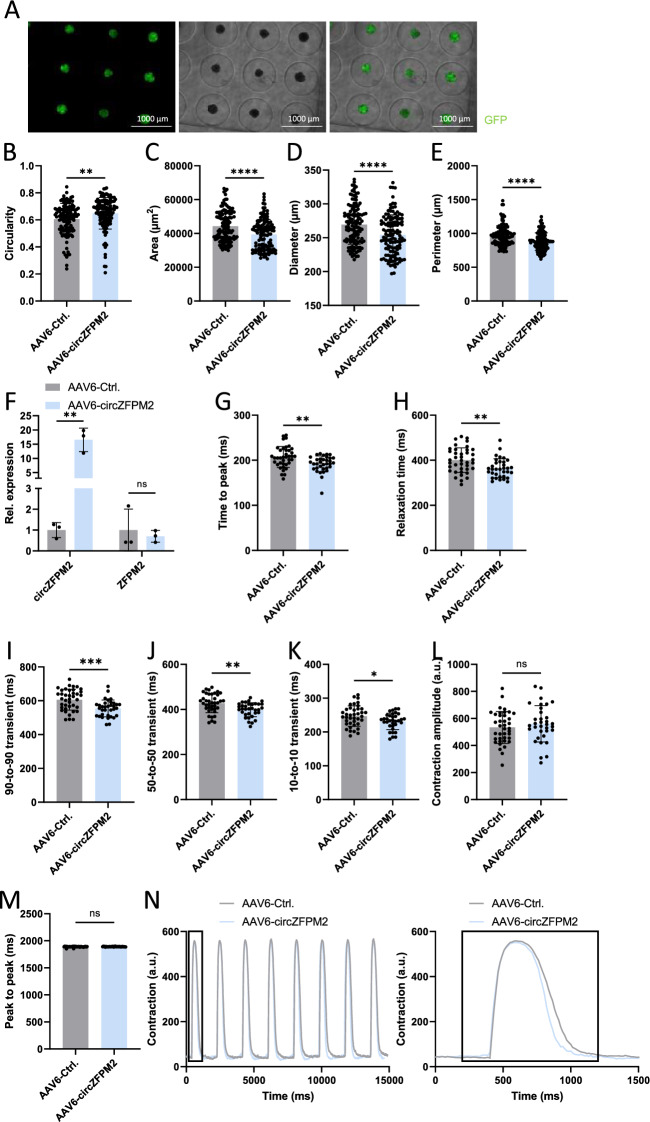
CircZFPM2 overexpression improves contractility in HCM-derived cardiac organoids. **A** Representative live-cell images of AAV6-GFP transduced human cardiac organoids (hCOs). **B**−**E** Morphological parameters of AAV6-circZFPM2 and AAV6-Ctrl. treated HCM-hCOs, displaying **B** circularity, **C** area, **D** diameter and **E** perimeter. Non-parametric t-test was performed to calculate significances (n = 123–124 hCOs/group). **F** Relative expression of circZFPM2 and ZFPM2 in AAV6-circZFPM2 and AAV6-Ctrl. treated HCM-hCOs. Non-parametric t-test was performed to calculate significances (n = 3 wells/group) **G**−**N** Contractile measurements of AAV6-circZFPM2 and AAV6-Ctrl. treated HCM-hCOs, displaying **G** time to peak, **H** relaxation time, **I** 90-to-90 transient, **J** 50-to-50 transient, **K** 10-to-10 transient, **L** contraction amplitude and **M** peak to peak time. Non-parametric t-test was performed to calculate significances (n = 34–39 hCOs/group). **N** Representative contractility plots of AAV6-circZFPM2 and AAV6-Ctrl. treated HCM-hCOs. Data are means ± SD. *p < 0.05; **p < 0.01; ***p < 0.001; ns = not significant

HCM-hCOs transduced with a GFP control virus exhibited homogenous distribution of GFP-positive HCM-CMs, illustrating a high transduction efficiency for our 3D AAV6 overexpression model (Fig. 8A). Morphologically, HCM-hCOs transduced with circZFPM2 exhibited improved circularity as well as reduced size, as evident by a reduced perimeter and diameter compared to control organoids (Fig. 8B–F). Functionally, HCM-hCOs overexpressing circZFPM2 showed improvement of several contractile parameters; reduced time-to-peak (Fig. 8G), relaxation time (Fig. 8H, N), 90-to-90 transient (Fig. 8I, N), 50-to-50 transient (Fig. 8J, N) and 10-to-10 transient (Fig. 8K, N). However, neither the contraction amplitude (Fig. 8L, N), nor the peak-to-peak time (Fig. 8M, N), were effected by elevated circZFPM2 levels in HCM-hCOs.

### CircZFPM2 signaling influences mitochondrial membrane potential and cardiomyocyte contractility

Finally, to decipher the role of circZFPM2 signaling, we performed RNA sequencing of total RNA from hiPSC-CMs, in which we silenced circZFPM2 with our backsplice site-specific siRNA.

**Fig. 9:**
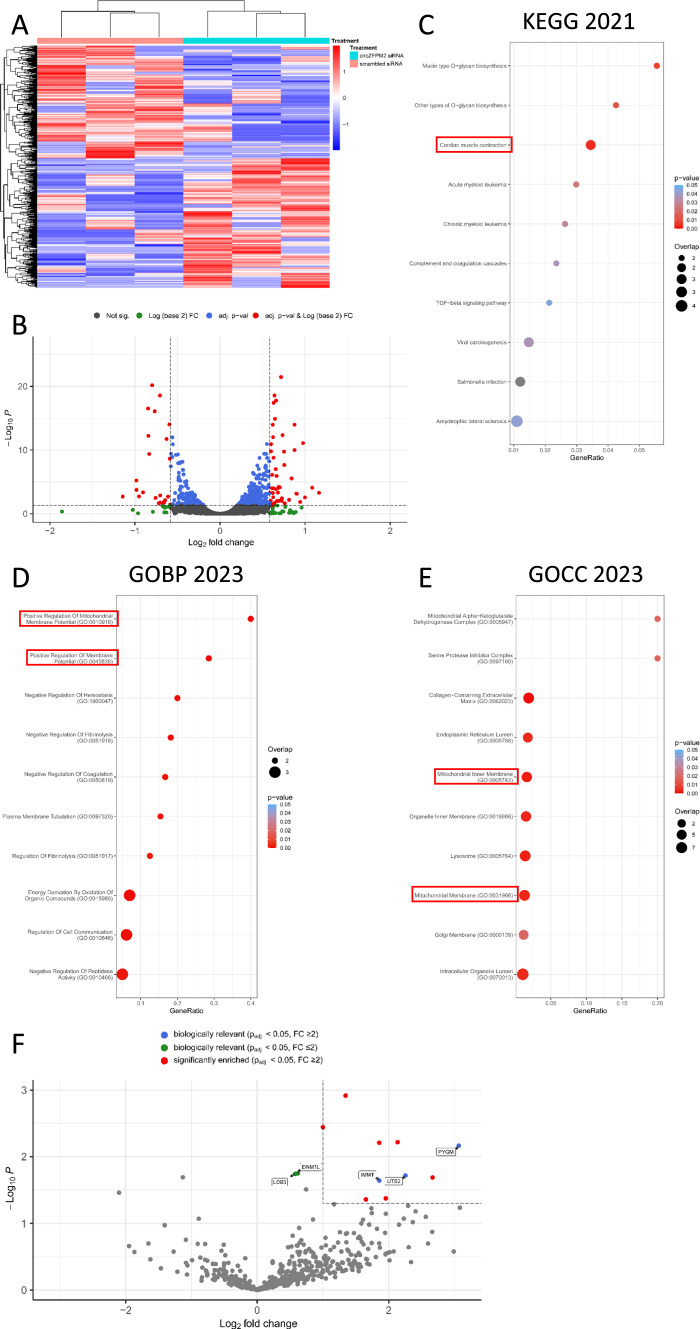
CircZFPM2 downstream signaling modulates mitochondrial function and cardiac muscle contractility. **A** Heat map of selected genes from the RNA sequencing dataset of circZFPM2 siRNA and control (scr.) siRNA treated hiPSC-CMs. Blue indicates significantly downregulated genes, red indicates significantly upregulated genes. **B** Volcano plot of the RNA sequencing dataset of circZFPM2 siRNA and scr. siRNA treated hiPSC-CMs. DEGs are highlighted in red. **C** KEGG 2021, **D** GOBP 2023 and **E** GOCC 2023 analysis of dysregulated gene sets from the RNA sequencing dataset. **F** Volcano plot of RNA pulldown samples from hiPSC-CM lysates against biotinylated circZFPM2 probe and scr. probe. Significantly enriched proteins with a FC ≥ 2 are highlighted in red. Potential interaction partners with biological relevance are highlighted in blue (FC ≥ 2) and green (FC < 2). FC, fold change

We observed 72 dysregulated genes (P_adj_ ≤ 0.05, FC ≥ 1.5) in circZFPM2-silenced hiPSC-CMs compared to scrambled siRNA (Fig. 9A, B). Gene set enrichment analysis using the KEGG database resulted in the enrichment of the “Cardiac muscle contraction” signaling pathway (Fig. 9C), and additionally, the GO terms, “Positive Regulation of Mitochondrial Membrane Potential” (GO:0010918), “Mitochondrial Inner Membrane” (GO:0005743) as well as “Mitochondrial Membrane” (GO:0031966) (Fig. 9D, E) were enriched in our RNA sequencing dataset. In line with our Seahorse data, which indicate impaired mitochondrial respiration (Fig. 3E, F and Fig. 5C–E), these results suggest a potential mode of action of circZFPM2 in HCM pathology via the modulation of oxidative phosphorylation in CMs. Furthermore, we aimed to identify potential interaction partners of circZFPM2 via RNA pulldown experiments. To do so, a probe complementary to the backsplice site of circZFPM2 and a scrambled probe as a control was designed and RNA pulldowns performed from hiPSC-CM cell lysates. Next, the samples were analyzed via mass spectrometry and peptide profiles were searched against human entries of uniprot database. From this dataset, we identified 4001 protein groups, of which 472 were quantified in circZFPM2 probe samples. Further analysis revealed 10 significantly enriched proteins (p_adj_ ≤ 0.05, FC ≥ 2) with PYGM, UTS2 and IMMT being the most meaningful candidate as potential interaction partners for circZFPM2 in CMs (Fig. 9F). PYGM is a key enzyme in glycogenolysis and thereby essential in providing sufficient energy for the contraction of muscle cells [33]. UTS2 has already been linked to several diseases, including cardiac fibrosis, cardiac hypertrophy, and heart failure [39]. It has been shown to effect cardiac contractility, cardiac hemodynamics, as well as induce cardiomyocyte hypertrophy [20, 39, 43]. IMMT is part of the MICOS-complex and located at mitochondrial cristae junctions [54]. Knockdown of IMMT results in structural damage of mitochondria, increased ROS production, and compromised oxidative phosphorylation [54, 60]. Moreover, despite not meeting the criteria of a fold change ≥ 2, other proteins involved in key mitochondrial or sarcomeric functions such as DNM1L and LDB3 were significantly enriched by our specific circZFPM2 probe. DNM1L contributes to mitochondrial fission, and muscle-specific loss-of-function experiments demonstrated functionally abnormal mitochondria, muscle wasting, and weakness [13, 50]. Further, LDB3 stabilizes sarcomere structures in cardiac muscle by binding α-actinin, and mutations within LDB3 have already been associated with various types of cardiomyopathies [4, 29, 55, 58].

Taken together, these data further suggest an effect of circZFPM2 on cardiac muscle contraction and mitochondrial function within cardiomyocytes in the context of HCM.

## Discussion

Although HCM represents the most common primary cardiac disorder, imposing a tremendous socioeconomic burden, limited specific treatment options are available [38]. Here, we present the circRNA circZFPM2 as a novel therapeutic target for the treatment of cardiac hypertrophy including HCM. We demonstrated that circZFPM2 activation protects from cardiomyocyte hypertrophy, improves cardiac metabolism and cellular health, as well as partially reverses the hypertrophic phenotype of patient-derived HCM-CMs.

While circZFPM2 in cardiovascular disease remains functionally uncharacterized to date, the linear host gene, ZFPM2, encodes a zinc finger protein that primarily interacts with the GATA family of transcription factors to regulate hematopoiesis and cardiogenesis in mammals [8, 30, 53]. Mutations in the ZFPM2 gene are embryonically lethal in mice [26]. This lethality seems to be attributed to the dysfunctional ZFPM2 protein, rather than circZFPM2, since mutations are mostly found in exons that do not comprise circZFPM2, and thus disrupt only ZFPM2 function [14]. Nevertheless, it is now established that circRNAs can exert host gene (protein)-independent functions [35, 45]. For example, we have recently demonstrated host gene-independent cardioprotective effects of a circular RNA derived from the insulin receptor locus in the context of doxorubicin-induced cardiotoxicity [31].

In order to select a novel circRNA candidate with potential clinical value, we performed RNA sequencing directly on heart tissue derived from HCM patients rather than assessing a hypertrophic animal model. CircZFPM2 was selected as the most promising candidate, since it successfully passed through stringent bioinformatic and experimental validation and also exhibits a high degree of species conservation including small (mouse, rat) and large (pig) animals, which constitutes an essential trait towards (pre)-clinical development. The heart-, and particularly cardiomyocyte-specific, expression pattern of circZFPM2 is a further desirable feature concerning its druggability and potential off-target effects in other cell types. Its expression increases with age, independent of ZFPM2 expression, suggesting a host gene independent function. Interestingly, the regulation of circZFPM2 in the disease context seemed to be specific to different models of cardiac hypertrophy, while its expression remained unaffected in other cardiac stress/disease conditions.

Initially, since circZFPM2 was significantly upregulated in HCM, we speculated whether its knockdown might alleviate the disease phenotype. Surprisingly, silencing of circZFPM2 in NRCMs induced cardiomyocyte hypertrophy and triggered metabolic alterations characteristic for HCM. CircZFPM2 knockdown led to enhanced ROS production, which is linked to cardiac hypertrophy [19, 40]. Hypertrophic cardiomyocytes generated higher ROS levels while ROS inducing agents caused an increase in cardiomyocyte size, emphasizing the linkage between mitochondrial performance and cardiac hypertrophy [41]. We also observed reduced cell viability concomitant with increased cytotoxicity, caspase activity, and apoptosis. While the precise underlying mechanism remains unclear, it is conceivable, that these phenotypes are consequences of the metabolic changes provoked by circZFPM2 loss, such as the increased ROS generation.

In contrast, circZFPM2 overexpression exerted beneficial therapeutic effects as indicated by partial rescue from LIF induced cardiomyocyte hypertrophy as well as from cytotoxicity and enhanced caspase activity after doxorubicin stress.

To functionally test circZFPM2 in a translational setting, we repeated the loss- and gain-of-function experiments in human iPSC-CMs. Importantly, the knockdown of human circZFPM2 showed similar but more pronounced adverse effects, possibly due to a more efficient siRNA knockdown compared to rodent cells. For the gain-of-function experiments in the human cells, we produced recombinant circZFPM2 as an alternative overexpression strategy using in vitro transcription (IVT) technology. This was based on two considerations; first, the therapeutic effect of the IVT RNA would be immediate compared to AAV. Second, despite the fact that AAV6-mediated delivery constitutes a reliable system, when using circZFPM2 overexpression cassettes the production of non-circularized RNA molecules cannot be avoided. Thus, using IVT circZFPM2 allowed us to pinpoint potential therapeutic effects specifically to the circular RNA. We used IVT and a complementary DNA splint for T4 ligase-mediated circularization to produce IVT circZFPM2, which had the correct size and backsplice site and which was stable after transfection into cells compared to the non-circularized control. Importantly, transfection of IVT circZFPM2 in hiPSC-CMs reproduced the results of AAV-mediated overexpression in rodent cells, i.e. decreased hypertrophic marker gene expression and alleviation from doxorubicin stress.

Encouraged by these results in healthy hiPSC-CMs, we used HCM-CMs as a disease model to further explore the clinical relevance of circZFPM2. Interestingly, circZFPM2 knockdown did not exert additional pro-hypertrophic effects, presumably owing to an already activated hypertrophy state resulting from the mutation in the *MYH7* gene. Nevertheless, circZFPM2 silencing significantly increased cytotoxicity and caspase activity. More importantly, the therapeutic effects of IVT circZFPM2 treatment were more pronounced in HCM-CMs compared to wild type iPSC-CMs, highlighting a potential clinical value of circZFPM2 therapy in the context of HCM.

In line with our 2D in vitro findings, our human 3D cardiac multi-cellular HCM cardiac organoid model revealed improved morphology and cardiac functional parameters after circZFPM2 overexpression. These beneficial effects included a reduced contraction duration as evident by reduced transient times upon AAV6-mediated circZFPM2 overexpression. Impaired relaxation is an early disease signs of HCM [16]. Interestingly, HCM-hCOs harboring our circZFPM2 overexpression construct, exhibited reduced relaxation time, indicating that circZFPM2 could have a favorable effect on pathological contractility in HCM. Mavacamten, an allosteric inhibitor of the cardiac myosin ATPase, improves clinical conditions of HCM patients by reducing contractility through the reduction of the formation of actin-myosin cross-bridges [36, 49]. However, Mavacamten also strikingly reduces the active contractility force in human engineered heart tissue [49] whereas circZFPM2 did not impair the contraction amplitude in HCM-hCOs. These results make circZFPM2 an interesting candidate to improve impaired cardiac muscle contractility in HCM.

Accordingly, transcriptome profiling of circZFPM2-deficient hiPSC-CMs revealed a significant regulation of the cardiac muscle contraction pathway. These results were further supported by our screening using RNA pulldown experiments, in which Urotensin-2, a vasoconstrictor, which also effects contraction and has been linked to cardiac hypertrophy [39], as well as LDB3, a stabilizer of sarcomere structures [4], were identified as potential protein interaction partners for circZFPM2. Furthermore, the RNA-seq dataset suggested a role of circZFPM2 in mitochondrial function according to the regulation of congruent GO terms, which could be verified by our Seahorse Mitochondrial Stress Test experiments, and is further supported by the potential interaction partners IMMT and DNM1L. IMMT is crucial for structural integrity of mitochondria and promotes oxidative phosphorylation while concurrently inhibiting ROS production [54, 60]. Oxidative phosphorylation was impaired upon circZFPM2 knockdown and circZFPM2 siRNA promoted intracellular ROS levels while IVT circZFPM2 reduced these, suggesting a cardioprotective mode of action of circZFPM2 via IMMT.

Altogether, the cardioprotective traits of circZFPM2 are conserved between species and transferable to human 2D as well as 3D HCM disease cell culture models, collectively proposing circZFPM2 overexpression therapy as a potential strategy to treat HCM.

### Limitations

Despite the demonstration of clear functional and therapeutic effects of circZFPM2 in cardiac hypertrophy, some limitations of the present study should be noted. Firstly, the precise underlying molecular mechanisms of circZFPM2 in cardiomyocytes remain unknown and therefore these beneficial therapeutic effects warrant further investigation. So far, our proteomics analysis from RNA pulldown experiments identified binding partners for circZFPM2 that are likely to mediate circZFPM2 beneficial effects, which have yet been verified in upcoming studies. Secondly, it was highly interesting that we observed stronger therapeutic effects of circZFPM2 in HCM patient-derived CMs. Although ideally, these experiments need to be performed with isogenic corrected HCM lines instead of in wild type hiPSC-CMs as the control group. Thirdly, despite using state of the art 2D in vitro cardiomyocyte models by utilizing NRCMs and hiPSC-CMs, as well as implementing a 3D model of HCM-derived human cardiac organoids, our study lacks information about the role of circZFPM2 in an HCM in vivo model. However, as the cardiac organoids are comprised of human cells, they add a translational aspect lacking in an in vivo mouse model, while maintaining the 3D structure and functional aspects of a whole heart organ system, with the added benefits of an observable HCM phenotype seen through the contraction measurements. Owing to the high sequence and functional conservation of circZFPM2, in vivo studies in large animals such as pigs are also eventually warranted for any future pre-clinical testing.

## Conclusion

CircZFPM2 is a cardiomyocyte-enriched circRNA that is specifically upregulated in HCM. Surprisingly, downregulation of this candidate showed pro-hypertrophic effects with impaired mitochondrial performance and cardiomyocyte health, whereas its further overexpression rescued cardiomyocyte function seen in 2D models across species, as well as in 3D human cardiac organoids. Thus, we conclude that circZFPM2 is upregulated in HCM in a compensatory and protective manner. Its further upregulation exerts beneficial effects in terms of hypertrophy, metabolism, and cytotoxicity, suggesting that circZFPM2 might serve as a promising treatment for urgently needed, novel HCM therapeutic approaches.

### Supplementary Information

Below is the link to the electronic supplementary material.

Supplementary file1 (PDF 1174 KB)
