# Supplementary material for: Circular RNA circZFPM2 regulates cardiomyocyte hypertrophy and survival
Source: Basic Res Cardiol. 2024 Apr 19;119(4):613–32. doi: 10.1007/s00395-024-01048-y (PMC11319402; doi:10.1007/s00395-024-01048-y)
Supplement: Supplementary file 1 — Supplementary file1 (PDF 1174 KB) [file 395_2024_1048_MOESM1_ESM.pdf]

Supplementary Material

Human tissue sampling

Heart tissue samples for RNA sequencing were obtained from 5 HCM patients after myectomy and 5 healthy donors. For qPCR validation, heart tissue samples from 18 HCM patients and 18 healthy donors were utilized. This study was conducted with the approval of the international ethics committee of the Hannover Medical School, Germany, and in accordance with the guidelines from the declaration of Helsinki and its amendments or comparable ethical standards.

Supplemental Table 1. Overview on human heart samples used for the RNA sequencing.

	**Total (n)**	**Males (n)**	**Females (n)**	**Sex unknown**	**Mean age ± SD**
**Control hearts**	5	3	2	0	53 ± 7
**HCM hearts**	5	2	1	2	35 ± 15

Supplemental Table 2. Overview on human heart samples used for the qPCR validation.

	**Total (n)**	**Males (n)**	**Females (n)**	**Mean age ± SD**
**Control hearts**	17	10	7	36 ± 12
**HCM hearts**	14	7	7	48 ± 19

Circular RNA sequencing and *in silico* validation

Total RNA isolated from cardiac tissue from 5 healthy donors and 5 HCM patients was tested for RNA integrity using the Agilent Bioanalyzer, after RNase R treatment and removal of ribosomal RNA. Then, according to the manufacturer's instructions of the Illumina Truseq kit, the RNA library was prepared. With the help of the Illumina Hiseq2000 at a read length of 100 bp, the paired-end sequencing was performed. After quality trimming, short reads were aligned to circRNA reference sequences from circBase (www.circbase.org) utilizing bowtie2 in sensitive mode. Raw read counts were then obtained by feature counts. To quantifiy the data and differentiate significant levels between HCM and healthy group the R package DESeq2 was utilized. The integrative genomics viewer [11] was used to display data.

Supplemental Table 3: Differentially regulated circRNAs in HCM tissues and their level of regulation

ID	gene symbol	Fold Change	P_{adj}
hsa_circ_0104196	DAPK2	54,2509	0,0235
hsa_circ_0001839	KDM4C	45,2022	0,0294
hsa_circ_0007292	ATP5C1	32,2020	0,0100
hsa_circ_0021516	GAS2	23,1654	0,0183
hsa_circ_0007334	MBOAT2	22,9116	0,0170
hsa_circ_0007612	ORC5	22,8646	0,0464
hsa_circ_0084143	HOOK3	20,7050	0,0295
hsa_circ_0005762	WDPCP	20,1277	0,0484
hsa_circ_0004846	SAMD4A	19,8648	0,0306
hsa_circ_0005562	ARHGEF10L	17,4984	0,0400
hsa_circ_0007518	NDUFB2	17,4438	0,0303
hsa_circ_0030883	CARKD	16,0484	0,0343
hsa_circ_0000081	ROR1	15,2074	0,0265
hsa_circ_0000963	VN1R1	15,0771	0,0494
hsa_circ_0009027	ZNF362	14,6807	0,0202
hsa_circ_0000705	CNOT1	14,2092	0,0376
hsa_circ_0003258	ZNF652	13,9426	0,0494
hsa_circ_0008937	COG3	13,5368	0,0269
hsa_circ_0004364	ZC3HC1	13,0499	0,0338
hsa_circ_0140214	DMD	12,7613	0,0173
hsa_circ_0121864	TMCC1	12,3525	0,0440
hsa_circ_0006376	HOOK3	12,3266	0,0332
hsa_circ_0004752	C1orf50	12,2203	0,0357
hsa_circ_0004182	CRIM1	12,1147	0,0397
hsa_circ_0014614	DAP3	11,2432	0,0261
hsa_circ_0005332	ZBTB20	9,8074	0,0149
hsa_circ_0001009	FANCL	8,7179	0,0402
hsa_circ_0001756	HIPK2	7,6669	0,0473
hsa_circ_0131935	MLIP	6,8488	0,0298
hsa_circ_0004502	YAF2	6,8236	0,0348
hsa_circ_0000061	SCMH1	6,6104	0,0000
hsa_circ_0000586	UBE3A	6,3695	0,0035
hsa_circ_0000300	MYBPC3	6,1378	0,0341
hsa_circ_0001118	NDUFA10	6,1112	0,0314
hsa_circ_0000754	SSH2	5,9463	0,0260
hsa_circ_0001498	WDR41	5,9243	0,0478
hsa_circ_0008521	PSEN1	5,7935	0,0381
hsa_circ_0001470	GOLPH3	5,4516	0,0358
hsa_circ_0017454	WDR37	5,3472	0,0300

hsa_circ_0007099	ABHD2	5,2755	0,0381
hsa_circ_0008500	DLG1	5,1835	0,0258
hsa_circ_0000139	GON4L	4,9317	0,0393
hsa_circ_0002566	ZNF680	4,7805	0,0345
hsa_circ_0001776	ESYT2	4,6907	0,0300
hsa_circ_0005600	YPEL2	4,6292	0,0243
hsa_circ_0078784	PSMB1	4,5838	0,0360
hsa_circ_0000914	FKBP8	4,4349	0,0419
hsa_circ_0006717	PAPOLA	4,3458	0,0007
hsa_circ_0111596	AKR7L	4,2760	0,0113
hsa_circ_0001788	PROSC	4,1942	0,0445
hsa_circ_0001060	UXS1	4,1858	0,0249
hsa_circ_0000284	HIPK3	4,1844	0,0074
hsa_circ_0003848	PSEN1	4,1723	0,0412
hsa_circ_0082096	ZNF800	4,1452	0,0261
hsa_circ_0000831	CEP192	4,0606	0,0466
hsa_circ_0000211	SFMBT2	3,9312	0,0230
hsa_circ_0072309	LIFR	3,9049	0,0348
hsa_circ_0005164	KAT6B	3,8045	0,0034
hsa_circ_0000072	OMA1	3,7988	0,0228
hsa_circ_0001900	CAMSAP1	3,7422	0,0479
hsa_circ_0140217	DMD	3,7039	0,0144
hsa_circ_0000672	CLEC16A	3,6890	0,0407
hsa_circ_0000896	FARSA	3,6745	0,0442
hsa_circ_0002490	FCHO2	3,5828	0,0058
hsa_circ_0001190	DYRK1A	3,4695	0,0282
hsa_circ_0000390	FGD4	3,4610	0,0447
hsa_circ_0003054	ATXN10	3,4447	0,0327
hsa_circ_0002346	CRIM1	3,3169	0,0220
hsa_circ_0007108	ZFX	3,2522	0,0246
hsa_circ_0008103	USP28	3,2251	0,0092
hsa_circ_0001861	GRHPR	3,2191	0,0247
hsa_circ_0079422	ICA1	3,1966	0,0001
hsa_circ_0001340	TMCC1	3,1283	0,0272
hsa_circ_0088633	GARNL3	3,0218	0,0215
hsa_circ_0131936	MLIP	2,8525	0,0080
hsa_circ_0066608	ST3GAL6	2,7104	0,0111
hsa_circ_0000099	AMY2B	2,6563	0,0142
hsa_circ_0083444	MTUS1	2,6494	0,0094
hsa_circ_0001017	XPO1	2,5839	0,0163
hsa_circ_0001130	TMX4	2,5381	0,0235

hsa_circ_0000219	FAM188A	2,4938	0,0394
hsa_circ_0002333	TCONS_l2_00000968	2,4746	0,0438
hsa_circ_0002590	UBE2K	2,4562	0,0002
hsa_circ_0002151	PDIA3	2,4201	0,0481
hsa_circ_0001423	AFF1	2,3424	0,0090
hsa_circ_0099549	FGD6	2,3312	0,0082
hsa_circ_0001811	STAU2	2,3098	0,0061
hsa_circ_0000471	N4BP2L2	2,3005	0,0137
hsa_circ_0006916	HOMER1	2,2746	0,0476
hsa_circ_0120050	SLC8A1	2,2567	0,0162
hsa_circ_0006156	FNDC3B	2,2463	0,0007
hsa_circ_0071041	GAB1	2,2159	0,0059
hsa_circ_0087357	UBQLN1	2,1552	0,0238
hsa_circ_0019223	PLCE1	2,1263	0,0053
hsa_circ_0007353	LEPROTL1	2,0218	0,0368
hsa_circ_0000915	FKBP8	2,0141	0,0227
hsa_circ_0008234	FOXP1	1,9642	0,0330
hsa_circ_0003380	**ZFPM2**	1,9345	0,0309
hsa_circ_0093546	CCDC7	1,9303	0,0185
hsa_circ_0109477	ZNF302	1,9209	0,0001
hsa_circ_0110400	GSTM4	1,8931	0,0185
hsa_circ_0006308	RICTOR	1,8745	0,0367
hsa_circ_0110859	GBAP1	1,8744	0,0268
hsa_circ_0139548	TMEM164	1,8658	0,0239
hsa_circ_0001247	ATXN10	1,7844	0,0465
hsa_circ_0050119	FKBP8	1,6258	0,0262
hsa_circ_0109538	ZNF585B	1,6244	0,0015
hsa_circ_0002080	C15orf38-AP3S2	1,5859	0,0384
hsa_circ_0112625	RYR2	1,5638	0,0237
hsa_circ_0000311	SDHAF2	1,5263	0,0234

Animal experiments

Male C57BL/6J WT mice at the age of 3-6 months were kept in a light-controlled room with a 12:12 light-dark cycle and had free access to standard mouse chow and water. Left coronary artery ligation was conducted as previously described to induce myocardial infarction (MI) [5]. In brief, the mice were anesthetized with 2% isoflurane, then intubated and artificially ventilated. A 1.5 cm incision was made into the skin at the mid-thorax parallel to the ribs. The left pectoralis muscle was cut off, the muscle layers were pulled aside and thoracotomy was conducted between the second and third rib. Then, the left coronary artery was ligated with 8-0 suture, 1mm distal to the left atrium. When the chest was closed again, the animals received 0.8 mg/mL Metamizole in drinking water up to 3 days after the surgery. Until recovery, mice were kept in warm cage and MI was confirmed 1 day after the operation by echocardiography. 6 weeks post-MI, animals were sacrificed. Experiments were approved by the local authorities at Hannover Medical School and Niedersächsisches Landsamt für Verbraucherschutz und Lebensmittelsicherheit (LAVES).

Cell culture based methods

Neonatal rat cardiomyocyte isolation and culture

Following the manufacturer's instructions of the Neonatal Heart Dissociation Kit (Miltenyi), cardiomyocytes were isolated from neonatal rat pups at the age of 0.5 to 3 days. Minimum Essential Medium (MEM, Bioconcept), supplemented with 1% penicillin/streptomycin (Gibco), 2 µg/mL vitamin B12 (Sigma), 100 nM bromdeoxyuridine (BrdU, Sigma) and 5% fetal bovine serum (FBS, Gibco) were used for cell maintenance for three days until experiments were conducted.

HL-1 cell culture

Mouse atrial cardiomyocyte-like HL-1 cells were cultured in Claycomb media (Sigma) supplemented with 10% FBS (Sigma), 1% penicillin/streptomycin, 2 mM L-glutamine (Sigma) and 0.1 mM norepinephrine (Sigma). Cell culture flasks and plates were coated with 5 µg/ml fibronectin in 0.02% gelatin and cells were kept at 37°C, 5% CO_2 and 95% air in a humidified incubator.

Wild type hiPSC culture

Wild type human induced pluripotent stem cells (hiPSCs) (hHSC_Iso4_ADCF_SeV-iPS2, alternative name: MHHi001-A) were maintained in supplemented StemMACS full medium (Miltenyi) at 37°C and 5% CO_2 on cell culture plates coated with Geltrex (Gibco) [4, 4]. Every 5 days the cells reached confluence and were

passaged with Versene (Gibco) in StemMACS full medium (Miltenyi) supplemented with 2 µM Thiazovivin (Selleckchem) in a ratio of 1:6. By modulation of the Wnt pathway as previously described, the hiPSCs were differentiated into cardiomyocytes and then purified and maintained for 40-60 days in culture until experiments were conducted [7].

HCM patient derived iPSC culture

The HCM-iPSC line was cultured on cell culture plates coated with Geltrex (Gibco) and supplemented mTeSR1 maintenance medium (StemCell) [8]. Every 5 days, the cells reached confluence and were passaged using Accutase (StemCell). HCM-iPSCs were differentiated into cardiomyocytes by modulation of the Wnt pathway as previously described, and then purified and maintained for 40-60 days in culture until experiments were conducted [7].

Human cardiac fibroblast maintenance

Human cardiac fibroblasts (HCFs) were cultured in Fibroblast Growth Medium 3 (FGM3, PromoCell). The cells were passaged using pre-warmed Trypsin and kept in a humidified incubator at 37°C with 5% CO_2. HCFs were used from passage four to eight for experiments.

HEK cell maintenance

Human embryonic kidney cells (HEK293) were cultured in DMEM media (Gibco) supplemented with 10% FBS and 1% penicillin/streptomycin and used to produce AAV6 viral particles. The cells were passaged using pre-warmed Trypsin/EDTA and were seeded with desired cell numbers onto the desired plates and kept in a humidified incubator at 37°C with 5% CO_2.

Cell treatments

Actinomycin D

NRCMs were treated with 2 µg/mL Actinomycin D (Sigma Aldrich) in MEM medium from 0 to 48 h.

Doxorubicin

HiPSC-CMs were treated with 1 µM doxorubicin in cardio-culture maintenance medium (RPMI GlutaMAX supplemented with 1X B-27) (Gibco) and NRCMs with 0.25 µM doxorubicin in MEM-medium for 48 h.

Hypoxia

For hypoxic conditions, NRCMs were kept for 72h in the incubator with 0.01% O_2. The control plate was incubated for 72h under normoxic condition (21% O_2).

Transforming growth factor β

HCFs were treated with 5 ng/mL of the recombinant human transforming growth factor β (TGFβ) or the vehicle (0.1% bovine serum albumin + 4 mM HCl) for 72 h.

Phenylephrine

NRCMs were treated with 100 µM phenylephrine in MEM medium with reduced FBS concentration (1% FBS) for 72 h.

Leukemia inhibitory factor

NRCMs were treated with 5 nM leukemia inhibitory factor in MEM medium with reduced FBS concentrations (1% FBS) for 48 h.

Endothelin-1 (ET-1)

HiPSC-CMs were treated with 50 µM ET-1 in cardio-culture maintenance medium (RPMI GlutaMAX supplemented with 1X B-27) (Gibco) for 48 h.

Modulation of gene expression

Knockdown of circZFPM2

The knockdown of circZFPM2 was achieved by using species-specific siRNA specifically binding the backsplice site without targeting the linear host gene, while scrambled siRNA was used as negative control. The HPLC purified siRNAs were purchased from Eurofins Genomics.

Hsa circZFPM2 siRNA: GGACCAGGGCCGCUUGAA

Rno circZFPM2 siRNA: ACUGGGAUGGACCAGGGCCG

Scrambled siRNA: AGGUAGUGUAAUCGCCUUG

SiRNA transfection

Scrambled or circZFPM2 siRNA (Eurofins) was transfected to NRCMs and hiPSC-CMs at a concentration of 100 nM using Lipofectamine 2000 (Life Technologies) in Opti-MEM medium (Gibco). The transfection mix was replaced by supplemented MEM medium after 5 h (NRCMs) and cardio-culture maintenance medium

(RPMI GlutaMAX supplemented with 1X B-27) (Gibco) after 12 h (hiPSC-CMs), respectively. For both species, further experiments were conducted 72 h after siRNA transfection.

Overexpression of circZFPM2

Upregulation of circZFPM2 was achieved by using rodent circZFPM2 overexpression plasmids (NRCMs), AAV6 viral particles harboring the overexpression plasmid (NRCMs) or by transfection of human IVT circRNAs (NRCMs and hiPSC-CMs).

Design of the overexpression plasmid

The overexpression plasmid carried the rodent exon 2 and exon 3 of the ZFPM2 locus, flanked by splice sites and circularization elements consisting of 100 bp of the endogenous intron 1 and the endogenous upstream Alu element and downstream by 100 bp of the endogenous intron 3 and the endogenous downstream Alu element. The plasmid additionally contained a 50 bp long sequence with several restriction enzyme sites upstream and downstream of the overexpression cassette. This construct was purchased from Invitrogen and delivered in a vector backbone, the delivery vector (pMA-RQ (AmpR)).

CircZFPM2 overexpression sequence:

TTATTTTTTTGAAATGAGATCTTGTTTAATAGCCCAGACTGACCTTAAATTCCCAGTCCTTTCTCACCTTGGAAATCA
AAACAACTCCGCTTAAAGGACTTTTATTTCATTACCTTCCAAGAATGTTTTTGGTACAGTGTCCCTGATTCTTTTTCA
AGGGCCGCTGGAAGATGCCATCGACGACGAGGAAGAAGAGTGTCCGGTGGAGGAAGCCGAAGTCATCTCGAAA
GGCGACTTTCCACTGGAGGGAAGCTTCCCCGCAGGCTTTGAGCCCGAAAATCTGAGCTGCGAAGACGTGGAGTTC
TTTTGTAACAAAGGTGATGACGAAGGAATCCAGGAGCCAGCAGAATCAGATGGGGACAGCCATTCAGACAAACC
AGGACAGCCTGGGGTAGAGACAGATGACTGGGATGGACCAG**GT**AGGACTGAGAGTACAGCATTCAACCCACTTT
AAATTTTATCAGGATTTCACTTTTTCGTTTAAATTCTACATGTAAGGAAGAGGTAAAGGCAATGTGATTTTTCGAGA
CAGGGTTTCTCTGTATAGCCCTGGCTGTCCTGAAGCTCACTTTGTAGACCTGGCTGGCCTCAAACTCAGAAATCTGC
CTGCCTCTGCCTCCCGAGTGCTGGGATTAAAGGCGTGTGGCACCATGCCCGACT

Blue regions are the endogenous upstream and downstream Alu elements. Black regions are 100 bp of the endogenous intron 1 and 3. Bold regions are splice sites, the grey region is exon 2 of circZFPM2 and the green region is exon 3 of circZFPM2.

Bacterial transformation

100 µL of Stbl3 *E.coli* was gently thawed on ice. Then, 1 ng of the overexpression cassette containing the delivery vector was added and the mix was incubated for an additional 30 min on ice. The heat-shock

approach was utilized for plasmid uptake, meaning that the mix was heated for exactly 90 sec at 42°C and then cooled down for a further 2 min on ice. Then, 100 µL of SOC outgrowth medium was added, and the sample was incubated at 37°C and 300 rpm for 60 min, spread on pre-warmed LB/ampicillin agar plates and incubated up-side-down overnight at 37°C.

Plasmid amplification and validation

Single *E.coli* colonies were picked from the LB/ampicillin agar plates after bacterial transformation and inoculated in 5 mL LB/ampicillin media overnight at 37°C and 225 rpm. Then, according to the manufacturer's instruction of the NucleoSpin Plasmid (NoLid) Mini kit, the plasmid DNA was isolated and Sanger sequencing verified the correct delivery vector.

Cloning of overexpression vector

For the AAV6 overexpression system, the overexpression cassette was cloned into the AAV MCS 1.3 vector. Therefore, both the delivery vector and the AAV vector were cut with the restriction enzymes EcoRI and XbaI. Gel electrophoresis (1% agarose) was conducted to detect bands of the expected size, which were cut out and extracted from the gel, using the QIAquick gel extraction kit. Correct vectors and inserts (verified by Sanger sequencing) were ligated with a molar ratio of 1:3 with the T4 DNA ligase and transformed into Stbl3 *E.coli*. Then they were amplified, isolated and validated as previously described.

Production of AAV6 vectors

Plasmid amplification

Stbl3 *E.coli* containing the empty AAV MCS plasmid (cell biolabs, #VPK-410) as negative control, the circZFPM2 overexpression plasmid or the helper plasmid PDP6rs with rep and cap genes necessary for AAV production, were each added to 5 mL LB media supplemented with Ampicillin. These bacterial suspensions were incubated for 16 h at 37°C and 225 rpm and then added to 600 mL LB media supplemented with Ampicillin (100 µg/mL). Using the NucleoBond Xtra Maxi EF Kit (Macherey-Nagel) according to the manufacturer's protocol, the plasmids were isolated after an additional 16 h (37°C, 225 rpm).

Plasmid validation

After isolation, the plasmids were validated by restriction enzyme digestions. The PDP6rs plasmid was cut with ClaI and KpnI (incubation for 2 h at 37°C), while AhdI (incubation for 2 h at 37°C) and SmaI (incubation

for 2 h at 25°C) were used for the overexpression plasmid. The correct digest pattern was verified by agarose gel electrophoresis before proceeding with viral production.

AAV6 production

HEK293T cells were seeded on cell culture dishes. After reaching 80% confluence, the cells were transfected with the empty AAV MCS 1.3 vector or the overexpression vector along with the PDP6rs helper plasmid and polythylenimine (PEI) as shown in the following table:

Transfection mix for AAV production.

For 10 plates	Stock conc.	Final amount	Volume
**DMEM Basal**			50 mL –(A+B+C)
**AAV construct (e.g., empty)**	1 µg/µL	100 µg (10µg/plate)	100 µL (A)
**AAV serotype (pPDP6rs)**	1 µg/µL	360 µg (36µg/plate)	360 µL (B)
**PEI MAX**	1 mg/mL	4X 460 µg (1:4 ratio)	180 µL (C)
**Total**			50 mL

After medium change, the cells were incubated for an additional 72 h. The supernatant was then collected and the cells were scraped from the dishes. The collected cells were centrifuged twice for 10 min at 1000 x g, pellets were collected, resuspended in lysis buffer and stored at -80°C. To collect viral particles secreted into the supernatant or contained in detached cells, the collected supernatant was also centrifuged for 10 min at 1000 x g. 25 mL 40% PEG8000 solution were added per 100 mL supernatant. The mix was stirred for 1 h at 4°C and then kept at 4°C overnight without stirring. The next day, the sample was centrifuged for 15 min at 4°C and 2,800 x g. The pellets were resuspended in lysis buffer. Then, the frozen cells were subjected to 3 thaw-freeze cycles and the resulting cell lysate was added to the lysate from the supernatant of the dishes and incubated with 1 M $MgCl_2$ (final 1 mM) and benzonase (final 250 U/mL). This mixture was lysed mechanically and centrifuged. AAV particles from the supernatant were isolated using an iodixanol (OptiPrep, Progen) gradient. After ultracentrifugation (rotor type 70Ti) for 70 min at 16°C and 63,000 rpm, the 40% iodixanol fraction containing the mature virus was collected and the viral particles were concentrated using Amacon100K columns (Millipore). 5 µL of the viral suspension were collected for gDNA isolation (DNeasy Blood and Tissue Kit) to calculate the viral titer via qPCR. The remaining viral suspension was aliquoted and stored at -80°C.

AAV6 transduction

NRCMs were transduced with AAV6-Ctrl. (empty plasmid) and AAV6-circZFPM2 using a multiplicity of infection (MOI) of $1x10^4$ for 72 h.

***In vitro* transcription of circRNA**

To produce the artificial human circZFPM2, the linearized human circZFPM2 sequence (exon 2 and 3 of the human ZFPM2 locus) was purchased from Invitrogen. The DNA template containing the T7 promoter for *in vitro* transcription was generated by PCR. The entire linearized circZFPM2 sequence was amplified using a primer pair of which the forward primer exhibited a T7 promoter sequence. According to the manufacturer's protocol of the QIAquick PCR Purification Kit (Qiagen), the amplicon was purified and then used for *in vitro* transcription with the RiboMax Large Scale RNA Production System (Promega) with a modified protocol. In brief, a mixture of 5-10 µg DNA template, 10 µL T7 RNA polymerase, 25 mM ATP/CTP/UTP, 5 mM GTP and 20 mM GMP was incubated for 2 h at 37°C while shaking. DNA templates were removed by DNase treatment with the ratio of 1U DNase/1µg DNA template. For RNA isolation, 700 µL Qiazol and 140 µL Chloroform were added to the samples, mixed well and centrifuged for 5 min at 4°C and 17,000 x g. After transferring the supernatants to new Eppendorf tubes, sodium acetate in a 1:10 ratio and the same volume of isopropanol were added and then centrifuged for 5 min at 4°C and 17,000 x g. Finally, precipitated RNA was washed with 100% ethanol and resuspended in 40 µL double-distilled, RNase-free water. In order to facilitate the circularization, DNA splints (20 bp in length) binding 10 bp upstream and 10 bp downstream to the backsplice site were incubated in a molar ratio of 1:3 with the produced RNA and the T4 DNA ligase for 1 h at 37°C. Then the circularized RNA was isolated as described above and validated using the 2100 Bioanalyzer (Agilent Technologies) and by Sanger sequencing of the PCR products generated by using divergent primers.

Hsa T7 IVT circZFPM2 fw primer: TAATACGACTCACTATAGGCCGCTTGAAGATGCCATT

Hsa IVT circZFPM2 rev primer: CTGGTCCATCCCAGTCGTCT

Hsa IVT circZFPM2 splint: TCAAGCGGCCCTGGTCCATC

IVT transfection

HiPSC-CMs and NRCMs were transfected with the i*n vitro* transcribed (IVT) circZFPM2 using Lipofectamine RNAiMAX. 2 µL RNAiMAX per 1 mL OptiMEM and 100-500 ng IVT circRNA per 1 mL OptiMEM were incubated separately for 5 min at room temperature, then mixed together and incubated for further 15

min. 20 µL/40 µL/200 µL of the transfection mix were added drop-wise per well of a 96-/48-/12-well plate and experiments were performed without additional medium change after 24 h, 48 h or 72 h.

Nucleic acid-based methods

RNA isolation

RNA was isolated with QIAzol (Qiagen) according to the manufacturer's protocol. RNA concentrations were measured using Take3 Plates and the Bio-Tek plate reader (Synergy HT).

cDNA synthesis

Using the Biozym cDNA Synthesis Kit with random hexamer primers according to the manufacturer's protocol, 500-1000 ng RNA were reverse transcribed.

Primer design

The bioinformatic tool Primer3 (https://bioinfo.ut.ee/primer3/) was used for PCR primer design and specificity was verified with the NCBI Primer Blast (https://ncbi.nlm.nih.gov/tools/primer-blast/). Divergent primer pairs were designed to bind upstream and downstream of the backsplice site and thereby amplifying the characteristic backsplice region of the circRNA.

Primer list.

Primer name	Forward primer	Reverse primer
**Rno-HPRT**	TCCTCCTCAGACCGCTTT	TTTTCCAAATCCTCGGCATAATG
**Rno-β-actin**	ACTCCTATGTAGGTGACGAGGC	GACGTTATGAGTCACACCGTCG
**Rno-circZFPM2**	AGCCATTCAGACAAACCGGG	ACTCCACTTCTTCACAGCTCA
**Rno-ZFPM2**	TAGTGGGAGGCAAAGAGAAGC	AGAGTGCTCCATGTCGCTTT
**Rno-BNP**	TCTGCTCCTGCTTTTCCTTA	GAACTATGTGCCATCTTGGA
**Rno-ANP**	GCCGGTAGAAGATGAGGTCA	GGGCTCCAATCCTGTCAATC
**Rno-MCIP**	AGCTCCCTGATTGCCTGTGT	TTTGGCCCTGGTCTCACTTT
**Rno-β-MHC**	GCCAACACCAACCTGTCCAAGTTC	TTCAAAGGCTCCAGGTCTCAGGGC
**Mmu-HPRT**	GCGTCGTGATTAGCGATGAT	TCCTTCATGACATCTCGAGCA
**Mmu-18S**	GTAACCCGTTGAACCCCATT	CCATCCAATCGGTAGTAGCG
**Mmu-circZFPM2**	TTCAGACAAACCAGGACAGCC	CCTTTCGAGATGACTTCGGC

**Mmu-ZFPM2**	CAGAGTAAACCCCGGCAGAT	AGCTCAGATTTTCGGGCTCA
**Mmu-ANP**	GCTTCGGGGGTAGGATTGAC	AGACCCCACTAGACCACTCA
**Mmu-MCIP**	CTGCACAAGACCGAGTT	TGTTTGTCGGGATTGG
**Mmu-β-MHC**	TCTCCTGCTGTTTCCTTACTTGCT	CAGGCCTGTAGAAGAGCTGTACTC
**Mmu-α-MHC**	GGTCCACATTCTTCAGGATTCTC	GCGTTCCTTCTCTGACTTTCG
**Mmu-Col1a2**	CAGAACATCACCTACCACTGCAA	TTCAACATCGTTGGAACCCTG
**Hsa-GUSB**	GACACCCACCACCTACATCG	CTTAAGTTGGCCCTGGGTCC
**Hsa-HPRT**	AGGACTGAACGTCTTGCTCG	GTCCCCTGTTGACTGGTCATT
**Hsa-TBP**	CCACTCACAGACTCTCACAAC	CTGCGGTACAATCCCAGAACT
**Hsa-circZFPM2**	ACAGCAGAATCAGATGGGGAC	TCGCAGCTCAGATTTTCAGGC
**Hsa-T7-IVT-circZFPM2**	TAATACGACTCACTATAGGCCGCTTGAAGATGCCATT	CTGGTCCATCCCAGTCGTCT
**Hsa-ZFPM2**	TGGGAGGCAAAGAGAAGCTG	AGATGAGGCAAGTCTTGGGC
**Hsa-BNP**	CAAGATGGTGCAAGGGTCTG	TTCCTCTTAATGCCGCCTCA
**Hsa-ANP**	ACTCCTCTGATCGATCTGCC	CCTCCCTGGCTGTTATCTTCA
**Hsa-MCIP**	GTGCCGGGCCAAATTTGAG	GCCAGGTGTGAGCTTCCTAT
**Hsa-β-MHC**	CCTGCTCTGTGTCTTTCCCT	ACTGCCATCTCCGAATCTCC
**Hsa-α-SMA**	CCTGACTGAGCGTGGCTATT	GATGAAGGATGGCTGGAACA
**Hsa-CTGF**	GTGTGCACCGCCAAAGAT	GTGTCTTCCAGTCGGTAAGC
**Hsa-LOX4**	CTGCCACACAGGGAATTCATAC	GTGTATCGGCAGCAGCTAGG
**Suscr-HPRT**	CCATCACATCGTAGCCCTCT	TATATCGCCCGTTGACTGGT
**Suscr-TBP**	ACGTTCGGTTTAGGTTGCAG	GCAGCACAGTACGAGCAACT
**Suscr-circZFPM2**	ATGGACCAGGGCCGCTAGA	TCCAGTGGAAAGTCCCCCTT

PCR and qPCR

Polymerase chain reaction (PCR) was conducted using the HotStarTaq Master Mix (Qiagen) and the Thermocycler Biometra Trio (Analytik Jena). Real-time qPCR was performed with the Absolute Blue qPCR SYBR Green Mix (Thermo Scientific), utilizing the QuantsStudio7 (ABI). For both methods, target specific primers were used and all experiments were performed according to the manufacturer’s instructions. The target gene expression levels were normalized to the house keeping genes hypoxanthine-guanine phosphoribosyl transferase (HPRT), TATA-binding protein (TBP) or 18S ribosomal RNA (18S) and analyzed via the ΔΔCT method.

Agarose gel electrophoresis

The size of DNA fragments after restriction enzyme digestion and of (q)PCR products was determined by agarose gel electrophoresis. Depending on the expected size of the fragments, a 1-2% agarose gel was prepared. Therefore, agarose was dissolved in TAE buffer with the help of a microwave and 4 µL/100 mL Midori Green (Nippon Genetics) were added to the slightly cooled solution, which was filled into a horizontal gel chamber. After gel polymerization, the DNA was mixed with 6x DNA gel loading dye (60% glycerol, 10 mM Tris-HCl pH 7.5, 0.3% bromophenol blue, 60 mM EDTA) and loaded together with the Quick-Load Purple 100 bp or 1 kb DNA ladder (NEB) onto the agarose gel and run for approximately 1 h in TAE buffer at 130 V. Gel bands were visualized and imaged with an UV light gel documentation system.

Gel extraction and sequencing

DNA was extracted from agarose gels using the QIAquick Gel Extraction Kit (Qiagen) according to the manufacturer's instructions. For Sanger sequencing, 30 µg of the purified DNA in a volume of 15 µL were mixed with 2 µL of either the forward or reverse primer (10 µM) and sent to Eurofins MWG Operon.

Subcellular fractionation

HiPSC-CMs were harvested using Trypsin, counted and $1x10^6$ cells were collected per 1.5 mL tube. They were each washed with 1 mL PBS and centrifuged for 5 min at 500 g at room temperature. The cell pellet was lysed in 175 µL cold RLN1 solution (50 mM Tris-HCl pH 8.0, 140 mM NaCl, 1.5 mM $MgCl_2$, 0.5% Igepal, 2 mM Vanadyl Ribonucleosid Complex) and, after 5 min of incubation on ice, centrifuged again for 2 min at 4°C and 300 x g. The supernatant, containing the cytoplasmic fraction, was transferred to a new tube and stored on ice. The pellet, containing the nucleic fraction, was resuspended in 175 µL of cold RLN2 solution (50 mM Tris-HCl pH 8.0, 500 mM NaCl, 1.5 mM $MgCl_{2,}$ 0.5% Igepal, 2 mM Vanadyl Ribonucleoside Complex), followed by incubation for 5 min on ice and centrifugation for 2 min at 16,400 x g at 4°C. Then, the supernatant was transferred to a new tube and stored on ice. The remaining pellet contained the chromatin-associated fraction. For RNA isolation, 1 mL QIAzol was added to each of the three fractions and vortexed for 15 sec. The RNA was isolated using the miRNeasy Mini Kit (Qiagen) according to manufacturer's instructions.

Conservation studies

We obtained the sequences of circZFPM2, which is annotated in human (hsa_circ_0003380) and mouse (mmu_circ_0000597), from circBase (www.circbase.org). Since circZFPM2 is not annotated in rat and pig, we put together the sequences from exon 2 and 3 of the ZFPM2 locus of these species, which we obtained from Ensemble (www.ensemble.org) and evaluated the sequence homology of the four species by the NCBI standard nucleotide BLAST (www.blast.ncbi.nlm.nih.gov) and validated it by qPCR using species-specific divergent primers that amplify the backsplice site. After gel electrophoresis, PCR products were sent for Sanger Sequencing (Eurofins Genomics).

Immunostaining of cardiac troponin T

NRCMs were cultured in a 48-well format at a density of 30,000 cells/well and fixed with 4% paraformaldehyde for 10 min. After 3 times of washing with 1x PBS for 5 min each, the cells were permeabilized with 0.1% Triton X-100 for 30 min at room temperature and non-specific binding sites were blocked with 5% donkey serum in 1x PBS (blocking buffer) for 30 min at room temperature. Afterwards, the cells were incubated with the primary antibody (cardiac troponin T, 1:500, Abcam; ab8295) in blocking buffer overnight at 4°C and after 3 washing steps, the secondary antibody (1:500, donkey anti-mouse Alexa Fluor 488) and Hoechst 33342 (1:2,000, Thermo Fisher) were added and incubated for 2 h at room temperature in the dark. Finally, after 3 further washing steps, fresh PBS was added to the plates, which were kept at 4°C until images were taken.

Seahorse extracellular metabolic flux assay

Cells used for Seahorse XF Mito Stress test were cultivated in XFe96 cell culture microplates. For NRCMs, which were seeded at a densitiy of 15,000 cells/well, the plates were coated with 1% gelatin. For hiPSC-CMs, seeded at a density of 50,000 cells/well, the plates were coated with Matrigel. After transfection of the NRCMs and hiPSC-CMs with the rodent and human circZFPM2-siRNA, respectively, for 72 h, the oxygen consumption rate (OCR) was measured using the Seahorse XF96 Analyzer. Cells were washed twice with assay medium (Agilent) and 180 µL assay medium were then added for pre-equilibration for 1 h at 37 °C in a non-CO_2 incubator. After acquiring the baseline (OCR), 2 µM oligomycin, 1 µM FCCP and 0.5 µM rotenone and antimycin A were injected. Three measurements were taken after injection of each compound. For normalization, the OCR values were normalized to cell count by staining nulcei with Hoechst 33342 (Thermo Fisher) and automated-counting using the Cytation 1 (Biotek).

Reactive Oxygen Species (ROS) Assay

The generation of cellular ROS in NRCMs and hiPSC-CMs was measured in a 96-well format using the DCFDA/H2DCFDA-Cellular ROS Assay Kit (Abcam) according to the manufacturer's instruction. In brief, cells were washed with pre-warmed PBS, stained with 20 µM DCFDA solution followed by an incubation step for 45 min at 37°C in the dark and washed once again with PBS. The positive control was treated with 110 µM TBHP in assay buffer. Finally, the plates were measured using the Cytation 1 at Ex/Em=485/535 nm for 6 h at 30 min intervals.

Viability Assay

Cell viability was assessed using the Cell Proliferation Reagent WST-1 (Roche). Therefore, NRCMs were seeded in 96-well plates with a density of 30,000 cells/well. On the day of the experiment, 10 µL Cell Proliferation Reagent WST-1 was added per well. After 80 minutes of incubation at 37°C, the absorbance was measured at 630 and 450 nm using the HT Synergy plate reader (Biotek).

Cytotoxicity Assay

NRCMs were cultivated with a density of 30,000 cells/well and hiPSC-CMs at a density of 20,000 cells/well, both in 96-well plates. Using the CytoTox 96 Non-Radioactive Cytotoxicity Assay kit (Promega) according to the manufacturer's protocol, the lactate dehydrogenase (LDH) levels were measured. In brief, 10µL of 10x Lysis Solution were added to the positive control, and incubated for 45 min at 37°C. Then, supernatant of each well was transferred to a new 96-well plate, equal volumes of CytoTox 96 Reagent were added and incubated for 30 min at room temperature. Finally, 100 µL of Stop Solution was applied to each well and the absorbance was measured at 490 nm with the HT Synergy plate reader (Biotek).

Caspase Assay

NRCMs and hiPSC-CMs were cultivated in 96-well plates with a density of 30,000 cells/well and 20,000 cells/well, respectively. The caspase activity was assessed with the Caspase-Glo 3/7 kit (Promega) following the manufacturer's protocol. Briefly, the 96-well plate and the Caspase-Glo 3/7 Reagent were equilibrated to room temperature. Then, 100 µL of the Caspase-Glo 3/7 Reagent was added to 100 µL of medium per well. After 30 (NRCMs) and 60 min (hiPSC-CMs) of incubation, 100 µL of each well were transferred to a white-walled 96-well plate. The luminescence was measured using HT Synergy plate reader (Biotek).

TUNEL

NRCMs and hiPSC-CMs were cultured in a 96-well format at a density of 30,000 cells/well. CTnT immunostaining as described above and the In Situ Cell Death Detection Kit (Roche) were combined and used according to the manufacturer's instruction. In brief, the cells were fixed with 4% paraformaldehyde for 10 min. After 3 washing steps with 1x PBS for 5 min each, they were permeabilized with 0.1% Triton X-100 for 2 min on ice and washed again 3 times with 1x PBS. The positive control was treated 20 min with 1 U DNase. Then, except the negative control, all samples were incubated with the TUNEL reaction mixture for 1 h at room temperature while shaking, followed by cTnT immunostaining and imaging using the Cytation 1.

Fabrication of human cardiac organoids

Human cardiac organoids (hCOs) were fabricated based on previous published protocols [1, 9, 10]: Agarose molds were formed using master micromolds (Merck). Three days prior to assembly of hCOs, HCM-hiPSC-CMs were transduced with either AAV6-GFP or AAV6-circZFPM2 particles at an MOI of 10^4. hCO cell suspension ($2x10^6$ cells/mL) was consistent of 50 % HCM-hiPSC-CMs (35 to 46 days after differentiation start) and 50 % non-CMs (p6-p10): HCFs (Promocell, C-12375), human umbilical vein endothelial cells (HUVECs, Lonza, C2519A) and adipose-derived stem cells (ADSCs, Lonza, PT-5006) in a ratio of 4:2:1. The cell suspension was seeded and incubated for 15 min in the agarose micromolds. Subsequently, organoid medium supplemented with 2 µM Thiazovivin (Selleckchem) was added. Ratiometric organoid medium was composed of 50% CM-specific medium (DMEM/F12 (1:1) (1x) + GlutaMAX™ (Life Technologies), 10% FBS (Life Technologies), 1% MEM NonEssential Amino Acids Sol. (Life Technologies), 1 % penicillin/streptomycin solution), 28.6% FGM-3 medium (Promocell), 14.3% EGM-2 (Lonza), and 7.1% ADSC medium (DMEM (1x) + GlutaMAX™, 10% FBS, 1% penicillin/streptomycin solution). Medium was changed every second day after production. Images were captured with a PlanFluor 4x 0.13/16.50mm PhL objective (Nikon) in a BZ-X810 inverted fluorescence microscope (Keyence) and processed with FIJI ImageJ [13]. Area, perimeter, diameter (Feret's) and circularity of the hCOs were analyzed using Fiji analyze particles with a prior set individual threshold.

Contractility measurements in hCOs

For contractility recordings (day 10 after production), hCOs were embedded in droplets of 10 mg/mL Geltrex for 30 min at 37°C in a 12-well plate. Afterwards fresh organoid medium was added and the hCOs were equilibrated at 37°C. Videos were acquired at an Olympus IX83 microscope with a 10x air objective (NA 0.4, Olympus) and a metal halide light source. Images were recorded with a cooled CCD camera (Orca-

R2, Hamamatsu, Photonics) while pacing with 0.5 Hz (MyoPacer, IonOptix). Videos of beating organoids were acquired for 1 min using CellSens Dimension (version 1.16, Olympus) software and afterwards processed to 15 sec videos that were analyzed using the ImageJ/Fiji software MUSCLEMOTION7 [12] and a customized R-script.

Dissociation of hCOs for RNA isolation

Prior to RNA isolation, hCOs were harvested on day 10 after production and digested with Collagenase Type II (700 U/mL, Worthington) in DPBS (Gibco #14160) for 30 min at 37°C. After 5 min centrifugation at 12000xg (4°C), the cell pellet was dissolved in QiAzol and RNA was extracted following the manufacturer's instructions.

Total mRNA sequencing

HiPSC-CMs were transfected with either circZFPM2 siRNA or scrambled siRNA as previously described and harvested after 48 h. Total RNA was isolated using the miRNeasy Mini Kit (Qiagen) and RNA integrity was assessed utilizing a 2100 Bioanalyzer (Agilent Technologies). Following, 500 ng of each RNA samples were shipped to Novogene for RNA sequencing and mRNA library (poly-A-enriched) was prepared utilizing the ''Novogene NGS RNA Library Prep Set (PT042)*'' (Novogene). In detail, mRNA was purified using poly-T oligo-attached magnetic beads and fragmented. Fragmented mRNA was reverse transcribed using random hexamer primers, followed by second strand cDNA synthesis. After performing, end repair, A-tailing, adapter ligation and size selection, the library was amplified via PCR and purified. Qubit was used to check the library, real-time PCR for quantification and bionalayzer to determine size distribution. Ultimately, the quantified library was pooled and sequenced on NovaSeq X Plus (150 bp paired-end sequencing strategy) according to effective library concentration and data amount. Differentially expressed genes (DEGs) between circZFPM2 siRNA and scrambled siRNA groups were calculated with the R package DESeq2 (v 1.42.0) using a two-factor design. DEGs were illustrated in a volcano plot, created with the EnhancedVolcano package (v. 1.20.0). Functional enrichment analysis was performed with the EnrichR web portal and results were plotted using ggplot2 (v 3.4.4) package in R.

RNA pulldown

10^6 hiPSC-CMs were washed with ice-cold DPBS, resuspended in cell lysis buffer (50 mM Tris pH 7 (Roth), 10 mM EDTA (Invitrogen), 0.1 % SDS (Roth), 1 mM DTT (Sigma), 100 U/ml RNasin® Ribonuclease Inhibitor (Promega), cOmplete™ Protease Inhibitor (Roche)) and incubated on ice for 10 min. Cells were

mechanically sheared using a dounce homogenizer and incubated on ice for 15 min. Cell debris were removed by centrifugation for 10 min at 14,000 x g and 4°C. 5% of the cleared lysate were directly frozen for RNA and protein and served as input control. The remaining lysate was mixed with two volumes of hybridization buffer (0.5 M NaCl (Sigma), 0.1 % SDS, 50 mM Tris pH 7, 1 mM EDTA, 15 % formamide (Ambion), 1 mM DTT, 100 U/ml RNasin® Ribonuclease Inhibitor, cOmplete™ Protease Inhibitor), 10 µg biotynilated DNA probe was added and incubated for 3 h. Meanwhile, 100 µl Dynabeads™ MyOne™ Streptavidin C1 (Invitrogen) were washed three times with bead wash buffer (5 mM Tris-HCl pH 7.5 (Roth), 0.5 mM EDTA, 1 M NaCl) on a magnet. The washed beads were washed twice in solution A (100 mM NaOH (Sigma), 50 mM NaCl) and once in solution B (100 mM NaCl). Beads were resuspended in 200 µl bead blocking solution (1 µg/µl BSA (Ambion), 1 µg/µl Yeast tRNA (Invitrogen)) and incubated for 2 h at 4°C. Blocked streptavidin beads were washed twice with lysis buffer and finally resuspended in lysis buffer. The washed and blocked beads were added to the cell lysate and incubated for 1 h. Following, the samples were washed five times with wash buffer (10 mM Tris-HCl pH 7.5, 10 mM KCl (Roth), 1.5 mM $MgCl_2$ (Merck), 150 mM NaCl, 5 mM DTT, 60 U/ml RNasin® Ribonuclease Inhibitor, cOmplete™ Protease Inhibitor) and three times with MS Buffer (10 mM Tris-HCl pH 7.5, 150 mM NaCl). Before the final washing step, samples were divided for RNA (20%) and protein (80%) analysis. The RNA sample was resuspended in Qiazol, followed by RNA isolation according to the manufacturer’s instructions and the protein sample was stored at -80°C.

scrambled-probe: 5’Biosg-AAAAGGTAGTGTAATCGCCTTGTT-3’

circZFPM2-probe: 5’Biosg-AAACATCTTCAAGCGGCCCTGGTCCATCCCAGT-3’

Mass spectrometry analysis

Protein samples from the RNA pulldown as well as the input controls were alkylated with 4% acrylamide (Applichem) for 30 min and separated on a 4-15% Mini-PROTEAN® TGX™ Precast Protein Gel (Bio-Rad). Proteins were stained by Coomassie Brilliant Blue G-250 (Thermo Fisher) for 45 min and the gel was destained by washing twice with ddH_2O. The gels were covered with ddH_2O and stored at 4°C until further processing. Each lane was cut into four pieces and protein samples were in gel digested using trypsin. Digested peptides were extracted and and analyzed by Orbitrap MS (Thermo Fisher) as described previously [6]. Raw data were processed using MaxQuant [2] as well as Perseus [3] and searched against reviewed human entries of uniprot database. Proteins were identified by a FDR ≤ 0.01 on protein and peptide level. Different protein loads were corrected by normalizing protein intensities to median values. Additionally, only proteins quantified circZFPM2-probe samples were considered for quantification. All

proteins identified in circZFPM2-probe samples were tested against a scrambled-probe as well as input to identify enriched proteins and putative binding partners.

Statistics

Data is presented as mean±SD. For all results, statistics were calculated using GraphPad Prism software. Significant differences between 2 groups with <6 data points per group were calculated using the non-parametric unpaired 2-tailed t test, while the parametric test was used for groups with >6 data points. The non-parametric 1-way ANOVA with post hoc test was used for ≥3 groups with <6 data points and parametric test with >6 data points per group. $P < 0.05$ was considered as statistically significant.

References

1. Arhontoulis DC, Kerr CM, Richards D, Tjen K, Hyams N, Jones JA, Deleon-Pennell K, Menick D, Bräuninger H, Lindner D, Westermann D, Mei Y (2022) Human cardiac organoids to model COVID-19 cytokine storm induced cardiac injuries. J Tissue Eng Regen Med 16:799–811. https://doi.org/10.1002/term.3327
2. Cox J, Mann M (2008) MaxQuant enables high peptide identification rates, individualized p.p.b.-range mass accuracies and proteome-wide protein quantification. Nat Biotechnol 26:1367–1372. https://doi.org/10.1038/nbt.1511
3. Cox J, Mann M (2012) 1D and 2D annotation enrichment: a statistical method integrating quantitative proteomics with complementary high-throughput data. BMC Bioinformatics 13 Suppl 16:S12. https://doi.org/10.1186/1471-2105-13-S16-S12
4. Haase A, Göhring G, Martin U (2017) Generation of non-transgenic iPS cells from human cord blood CD34+ cells under animal component-free conditions. Stem Cell Res 21:71–73. https://doi.org/10.1016/j.scr.2017.03.022
5. Hobuß L, Foinquinos A, Jung M, Kenneweg F, Xiao K, Wang Y, Zimmer K, Remke J, Just A, Nowak J, Schmidt A, Pich A, Mazlan S, Reamon-Buettner SM, Ramos GC, Frantz S, Viereck J, Loyer X, Boulanger C, Wollert KC, Fiedler J, Thum T (2020) Pleiotropic cardiac functions controlled by ischemia-induced lncRNA H19. J Mol Cell Cardiol 146:43–59. https://doi.org/10.1016/j.yjmcc.2020.07.001
6. Jochim N, Gerhard R, Just I, Pich A (2011) Impact of clostridial glucosylating toxins on the proteome of colonic cells determined by isotope-coded protein labeling and LC-MALDI. Proteome Sci 9:48. https://doi.org/10.1186/1477-5956-9-48
7. Kreutzer FP, Meinecke A, Mitzka S, Hunkler HJ, Hobuß L, Abbas N, Geffers R, Weusthoff J, Xiao K, Jonigk DD, Fiedler J, Thum T (2022) Development and characterization of anti-fibrotic natural compound similars with improved effectivity. Basic Res Cardiol 117:9. https://doi.org/10.1007/s00395-022-00919-6
8. Merkert S, Wunderlich S, Beier J, Franke A, Schwanke K, Göhring G, Kraft T, Francino A, Zweigerdt R, Martin U (2021) Generation of two iPSC clones (MHHi021-A and MHHi021-B) from a patient with hypertrophic cardiomyopathy with p.Arg723Gly mutation in the MYH7 gene. Stem Cell Res 52:102208. https://doi.org/10.1016/j.scr.2021.102208
9. Richards DJ, Coyle RC, Tan Y, Jia J, Wong K, Toomer K, Menick DR, Mei Y (2017) Inspiration from heart development: Biomimetic development of functional human cardiac organoids. Biomaterials 142:112–123. https://doi.org/10.1016/j.biomaterials.2017.07.021
10. Richards DJ, Li Y, Kerr CM, Yao J, Beeson GC, Coyle RC, Chen X, Jia J, Damon B, Wilson R, Starr Hazard E, Hardiman G, Menick DR, Beeson CC, Yao H, Ye T, Mei Y (2020) Human cardiac organoids for the modelling of myocardial infarction and drug cardiotoxicity. Nat Biomed Eng 4:446–462. https://doi.org/10.1038/s41551-020-0539-4
11. Robinson JT, Thorvaldsdottir H, Turner D, Mesirov JP (2023) igv.js: an embeddable JavaScript implementation of the Integrative Genomics Viewer (IGV). Bioinformatics 39. https://doi.org/10.1093/bioinformatics/btac830
12. Sala L, van Meer BJ, Tertoolen LGJ, Bakkers J, Bellin M, Davis RP, Denning C, Dieben MAE, Eschenhagen T, Giacomelli E, Grandela C, Hansen A, Holman ER, Jongbloed MRM, Kamel SM, Koopman CD, Lachaud Q, Mannhardt I, Mol MPH, Mosqueira D, Orlova VV, Passier R, Ribeiro MC, Saleem U, Smith GL, Burton FL, Mummery CL (2018) MUSCLEMOTION: A Versatile Open Software Tool to Quantify Cardiomyocyte and Cardiac Muscle Contraction In Vitro and In Vivo. Circulation research 122:e5-e16. https://doi.org/10.1161/CIRCRESAHA.117.312067

13. Schindelin J, Arganda-Carreras I, Frise E, Kaynig V, Longair M, Pietzsch T, Preibisch S, Rueden C, Saalfeld S, Schmid B, Tinevez J-Y, White DJ, Hartenstein V, Eliceiri K, Tomancak P, Cardona A (2012) Fiji: an open-source platform for biological-image analysis. Nature methods 9:676–682. https://doi.org/10.1038/nmeth.2019

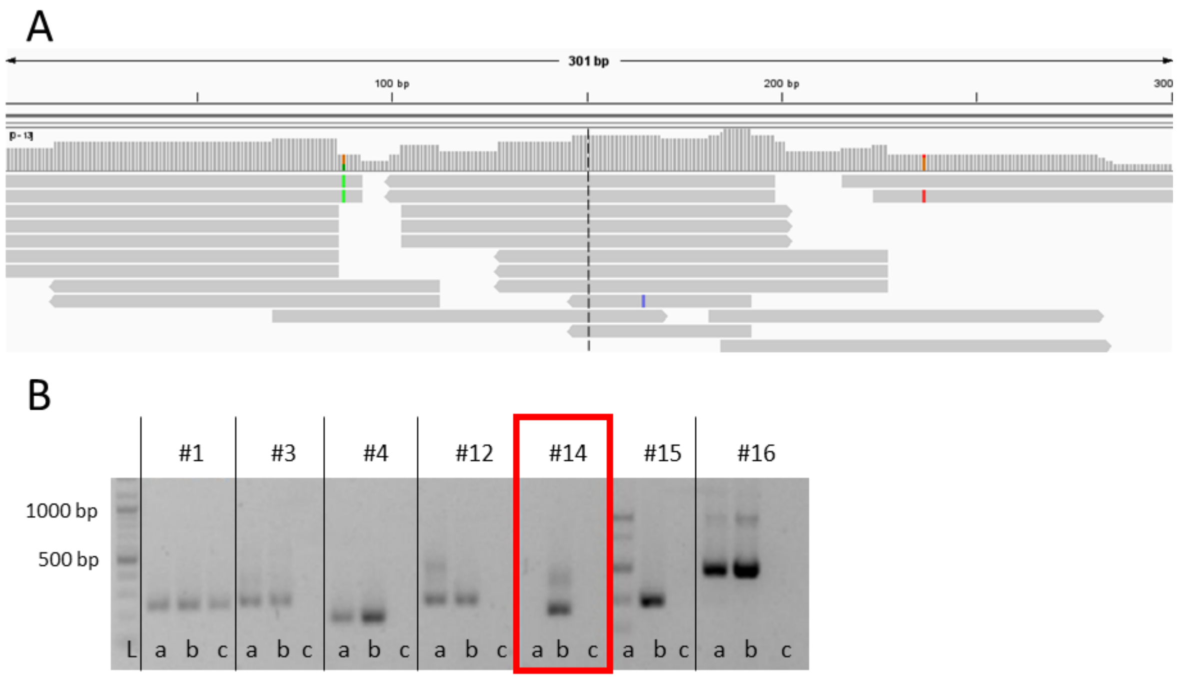

Supplementary Figure S1: ***In silico*** **validation of backsplice site and experimental selection of circZFPM2 as lead candidate. A** Integrative Genmocs Viewer (IGV) displays paired-end reads for an annotated circRNA. Dashed line represents the backsplice site. **B** Expression analysis of selected circRNA candidates via agarose gelelectrophoresis. Lanes with circZFPM2 are highlighted by a red box. #1: hsa_circ_0000061; #3: hsa_circ_0006156; #4: hsa_circ_0006156; #12: hsa_circ_0066608; #14: hsa_circ_0003380; #15: hsa_circ_0001776; #16: hsa_circ_0072309; L: ladder; a: hiPSC cDNA; b: hiPSC-CM cDNA, c: water.

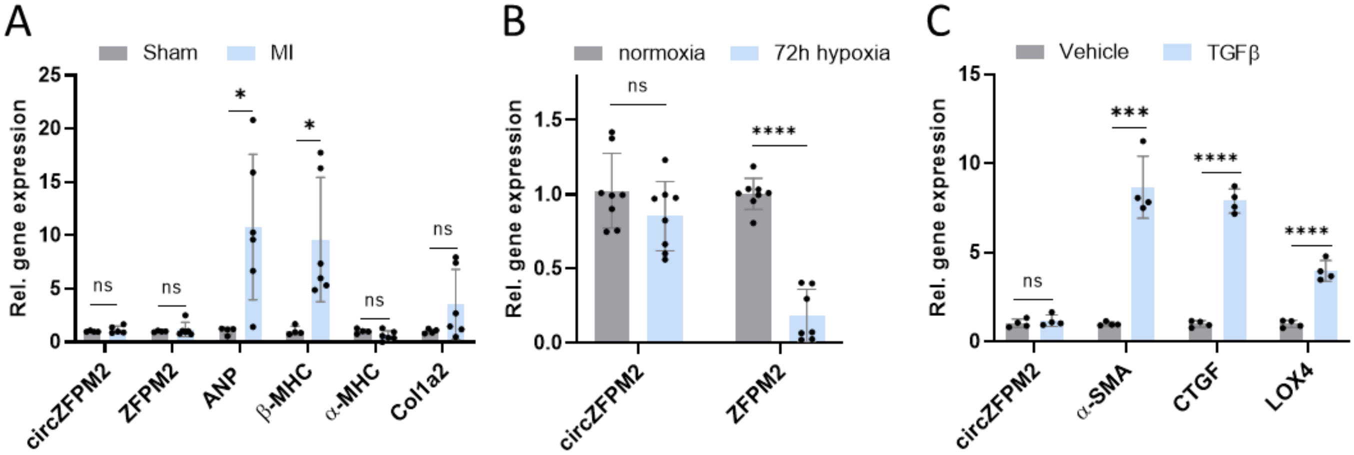

Supplementary Figure S2. A Relative expression of circZFPM2, ZFPM2, hypertrophy and fibrosis marker genes in a murine model of MI. Parametric t-test was performed to calculate significances (n = 6 mice/group). Sham, sham surgery; MI, myocardial infarction. **B** Relative expression of circZFPM2 and ZFPM2 in NRCMs under normoxic and 72 h hypoxic conditions (0.01% O_2). Non-parametric t-test was performed to calculate significances (n = 4-5 wells/group). **C** Relative expression of circZFPM2 and fibrosis marker genes in vehicle and 72 h TGFβ treated human cardiac fibroblasts. Non-parametric t-test was performed to calculate significances (n = 4 wells/group). TGFβ, transforming growth factor β. Data are means ± SD. *$p < 0.05$; ***$p < 0.001$; ****$p < 0.0001$; ns = not significant.

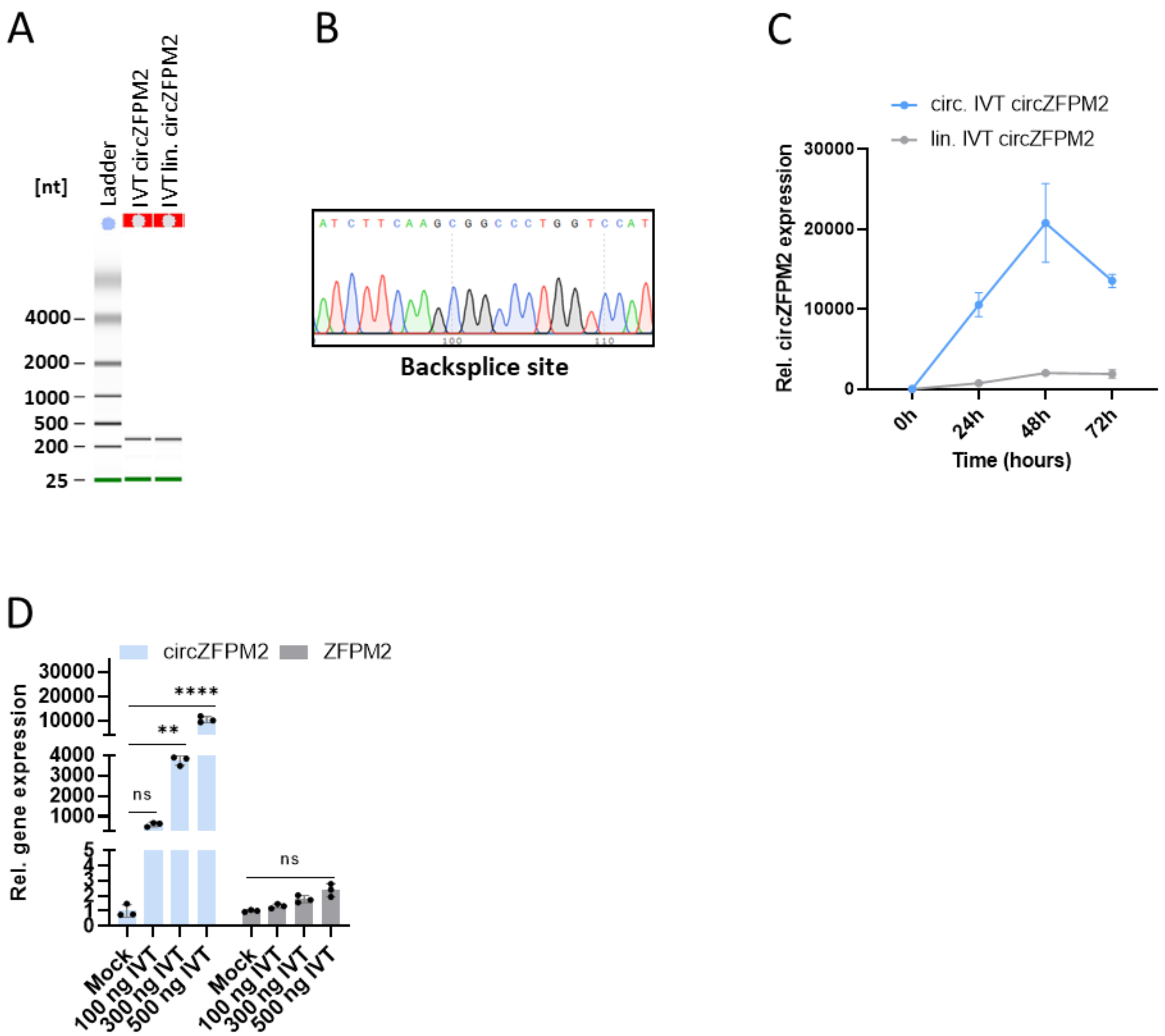

Supplementary Figure S3. *In vitro* transcription and validation of artificial circZFPM2. A Validation of the molecular size of the *in vitro* transcribed (IVT) linear and circular circZFPM2 using the BioAnalyzer. **B** Sanger sequencing results showing the backsplice site of the circularized *in vitro* transcribed circZFPM2 after PCR and gel electrophoresis. **C** Time line of the relative expression of *in vitro* transcribed linear and circular circZFPM2 (n = 3 wells/group) in hiPSC-CMs transfected with the linear and the circular IVT circZFPM2. **D** Relative expression of circZFPM2 and ZFPM2 in hiPSC-CMs treated with different concentrations of IVT circZFPM2. Non-parametric one-way ANOVA was performed to calculate significances to the respective Mock-control (n = 3 wells/group). Data are means ± SD. **p < 0.01; ****p < 0.0001; ns = not significant.
